# A-ZIP53, a dominant negative reveals the molecular mechanism of heterodimerization between bZIP53, bZIP10 and bZIP25 involved in *Arabidopsis* seed maturation

**DOI:** 10.1038/s41598-017-14167-5

**Published:** 2017-10-30

**Authors:** Prateek Jain, Koushik Shah, Nishtha Sharma, Raminder Kaur, Jagdeep Singh, Charles Vinson, Vikas Rishi

**Affiliations:** 1 National Agri-Food Biotechnology Institute, Knowledge City, Sector 81, Mohali, Punjab 140306 India; 2 Department of Biotechnology, Panjab University, Sector 14, Chandigarh, 160014 India; 3 Laboratory of Metabolism, National Cancer Institute, National Institutes of Health, Bethesda, 20892 USA

## Abstract

In *Arabidopsis*, maturation phase, an intricate process in seed formation is tightly regulated by the DNA binding activity of protagonist basic leucine zipper 53 (bZIP53) transcription factor and its heterodimerizing partners, bZIP10 and bZIP25. Structural determinants responsible for heterodimerization specificity of bZIP53 are poorly understood. Analysis of amino acid sequences of three bZIPs does not identify interactions that may favor heterodimerization. Here, we describe a designed dominant negative termed A-ZIP53 that has a glutamic acid-rich amphipathic peptide sequence attached to N-terminal of bZIP53 leucine zipper. Circular dichroism (CD) and mass spectrometry studies with equimolar mixture of three bZIP proteins in pairs showed no heterodimer formation whereas A-ZIP53 interacted and formed stable heterodimers with bZIP53, bZIP10, and bZIP25. A-ZIP53 electrostatically mimics DNA and can overcome repulsion between basic DNA binding regions of three bZIP proteins. Gel shift experiments showed that A-ZIP53 can inhibit the DNA binding of three proteins. CD studies demonstrated the specificity of A-ZIP53 as it did not interact with bZIP39 and bZIP72. Transient co-transfections in *Arabidopsis* protoplasts showed that A-ZIP53 inhibited three bZIPs and their putative heterodimers-mediated transactivation of GUS reporter gene. Furthermore, four newly designed acidic extensions were evaluated for their ability to interact with three bZIPs.

## Introduction

bZIP transcription factors (TFs) are eukaryote specific proteins that bind to short but specific DNA sequences as a dimeric parallel coiled coil and regulate gene expression. The bZIP domain is a long, bipartite alpha helix. There is a N-terminal DNA binding domain and a C-terminal coiled coil termed the leucine zipper containing leucine at the *d* position of the heptad (*g,a,b,c,d,e,f*)_n_ repeats^1–3^. bZIP TFs also have transactivation domain present either at N- or C-terminal^1^. These TFs homo- or heterodimerize *via* a monomer or dimer pathway and occupy major grooves of DNA^4–7^. Extensive biochemical and biophysical studies have established the rules that dictate bZIP TFs homo- and heterodimerization propensity. Genome-wide studies involving *Drosophila*, Human, and *Arabidopsis* have revealed that many features that are critical in specifying dimerization properties of bZIPs are conserved across the kingdoms^8–10^. For example, similar amino acids in *g*, *e, a*, and *d* positions in the heptads of all bZIPs suggest their common role in dimerization specificity.

Previously we have used the structural determinants in coiled coil motif to design dominant negative proteins called A-ZIPs and A-HLH against various bZIPs and structurally related basic helix loop helix (bHLH) TFs. Earlier studies have shown the efficacies of A-ZIPs in understanding the roles of bZIPs in human pathologies like cancer and diabetes^11–14^. In plants bZIP proteins are important for pathogen defense, light-induced signaling, flower development, and seed maturation^15^. In *Arabidopsis*, seed maturation is regulated by combinatorial effects of multiple bZIP TFs including bZIP53 and its heterodimerizing partners bZIP10 and bZIP25^16^. In *Arabidopsis*, numbers of studies have reported the heterodimerizing network of group C /S1 bZIPs proteins involved in seed development and maturation but only a subset of these bZIPs are identified in this context^15,17–20^. Analysis of amino acids sequences of bZIP10 and bZIP25 predicted that these are 8 heptad long and have amino acids in *g*, *e*, *a*, and *d* positions that favor homodimerization whereas bZIP53 is >8 heptad long with complex dimerization specificity^10^. Transient co-transfection studies, yeast two-hybrid system, and bimolecular fluorescence complementation (BiFc) assay, with bZIP53, bZIP10, and bZIP25 suggested heterodimer formations^18,21–23^. However, structural signatures that mediate heterodimerization between the three bZIPs are not well-understood.

Here, we describe A-ZIP53, a DNA binding inhibitor of bZIP53 with a designed acidic extension that mimic DNA. A-ZIP53 contains a rationally designed glutamic acid-rich peptide extension that replaces the N-terminal basic DNA binding domain of bZIP53. *In vitro* studies show that A-ZIP53 can heterodimerize with bZIP53, bZIP10 and bZIP25. Additionally, four derivatives of A-ZIP53 with different dimerization potentials were produced. Since A-ZIP53 and its four derivate can heterodimerize with bZIP53, bZIP10 and bZIP25 and inhibit their DNA binding, these proteins may be used to study dimerization specificity of these bZIPs *in vitro* and *in vivo*.

## Results

### Amino acids sequences of bZIP proteins and their propensity to form heterodimers

**Figure 1:**
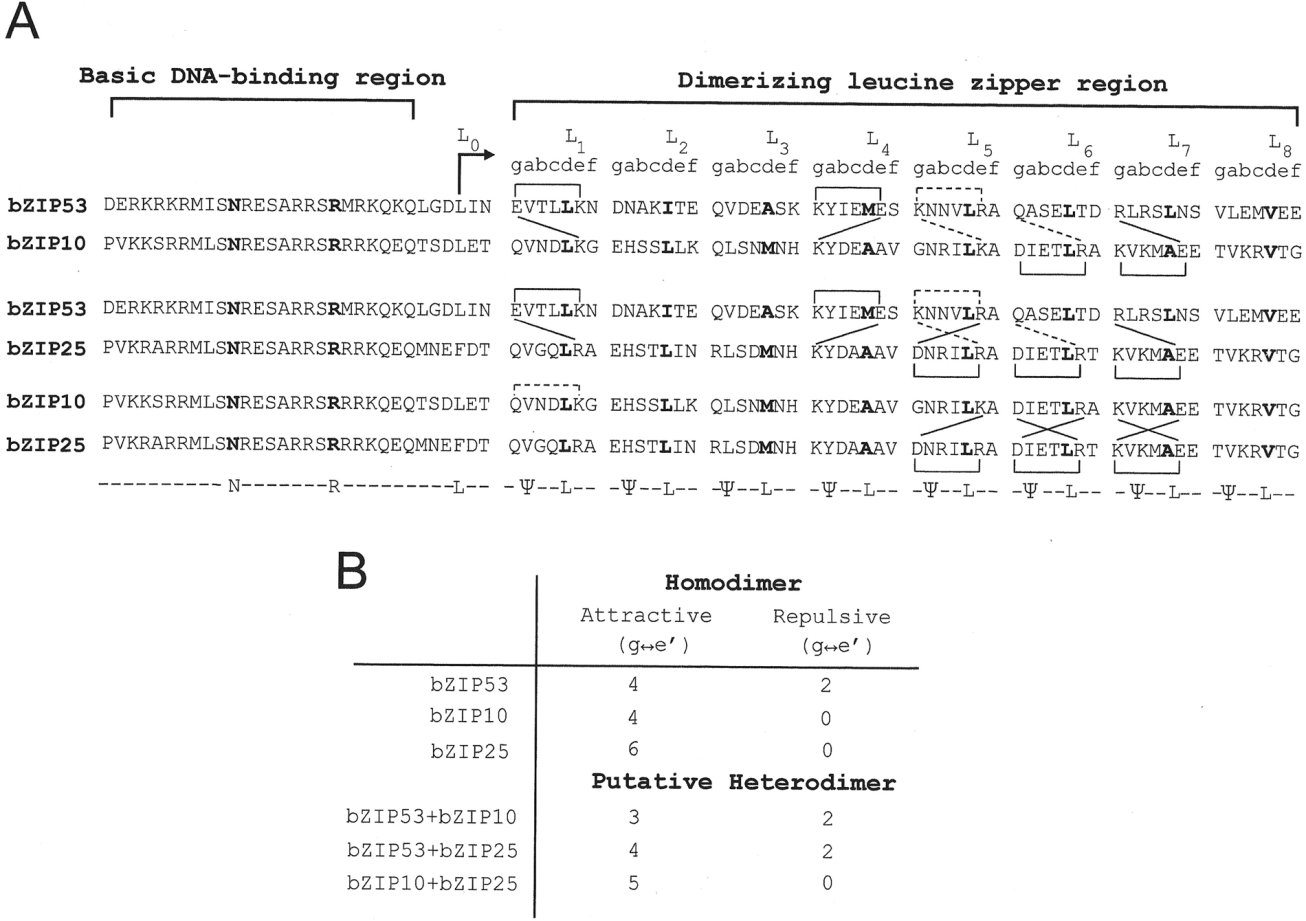
Amino acid sequences of bZIP53, bZIP10, and bZIP25. (**A**) At the top is the delineation of N-terminal basic DNA-binding region followed by dimerizing leucine zipper region. Amino acid sequences represented by the single-letter code are aligned with respect to an invariant asparagine (N) and arginine (R) (shown in bold) in the basic region. Only ten amino acids upstream of asparagine are shown. Tenth amino acid (typically a leucine; L_o_) from invariable arginine in the basic DNA-binding region marks the start of the dimerizing leucine zipper. The leucine zipper sequence is grouped into heptad (*a,b,c,d,e,f*, *g*)_n=8_. The limit of a coiled coil at C-terminus is defined by the presence of a proline or two consecutive glycines, both likely helix-breaking residues and the absence of charged amino acids in *g* and *e’* positions in a heptad. Also shown below is a consensus sequence for bZIP motif, where Ψ represents any hydrophobic amino acid. Proteins are placed in three groups with three homodimers and three potential heterodimers. In homodimer coiled coil, interhelical interactions between amino acids in the *g* position with those in the following *e*’ position are shown as square brackets. Solid square brackets depict attractive interactions between amino acids with opposite charges in *g* and *e*’ positions (E ↔ K, K ↔ E, D ↔ R) whereas interhelical repulsive interactions between *g* and *e*’ position amino acids are shown by discontinuous square brackets (K ↔ R). In the putative heterodimers coiled coil (bZIP53 + bZIP10, bZIP53 + bZIP25 and bZIP25 + bZIP10) attractive interactions between amino acids at *g* and *e*’ positions are shown by solid diagonal lines (E ↔ K, R ↔ E, R ↔ D, K ↔ D) and repulsive interactions are depicted by discontinuous lines (K ↔ K,K ↔ R). (**B**) The number of attractive and repulsive *g* ↔ *e*’ salt bridges formed in three homodimers and three putative heterodimers.

Charged amino acids in the *g* and *e*’ positions of coiled coil produce interhelical *g* ↔ *e*’ or i, i + 5 interactions. Presence of opposite or like charged amino acids in *g* and *e*’ positions promote or inhibit dimerization. In order to quantify the interactions that may favor homo- or heterodimerization among bZIP53, bZIP10 and bZIP25 we aligned and analysed their amino acids sequences. Figure 1 shows the amino acids sequences of bZIP53, bZIP10, and bZIP25. A single alphabet code is used to depict each amino acid. Sequences are aligned with respect to an invariant N (bold) in the basic region domain. Coiled coil nomenclature with each heptad represented by *gabcdef* is shown at the top of the leucine zipper dimerization domain. bZIP25 and bZIP10 are predicted to be 8 heptads long with their C-terminus boundaries defined by the absence of proline. bZIP53, due to the absence of a proline and two consecutive glycines, presence of charged amino acids in *g* and *e* position, and hydrophobic amino acids in *a* and *d* positions is predicted to be more than 8 heptads long. Three bZIPs can form homodimers and three potential heterodimers. In an earlier study, 67 bZIPs of *Arabidopsis thaliana* were placed in 20 families (A-T) based on their predicted dimerizing properties^10^. bZIP25 and bZIP10 were included in G family whereas bZIP53 was placed in H family. Presence of N in *a* position of 5^th^ heptad of bZIP25 and bZIP10 and three attractive *g* ↔ *e*’ (i, i’ + 5) interactions in 5^th^, 6^th^, and 7^th^ heptads of bZIP25 and two attractive interactions in 6^th^ and 7^th^ heptads of bZIP10 favor homodimerization. bZIP53 with N in *a* position of 2^nd^ and 5^th^ heptad and attractive *g* ↔ *e*’ interactions in 4^th^ heptad should favor homdimerization but repulsive *g* ↔ *e*’ interactions in 5^th^ heptad may initiate heterodimerization with other bZIP group members. Such a complex arrangement of amino acids makes it difficult to predict bZIP53 dimerization properties. Also shown are the amino acids sequences of three bZIPs in putative heterodimeric conformations. A quantification of the presumed electrostatic interactions between two proteins due to charged amino acids in *g* and *e* position of heptads is indicated in Fig. 1B. Since more attractive interactions are in homodimer coiled coil compared to heterodimers, the former will be a preferred conformation in an equimolar mixture.

### Thermal Stability of bZIP53, bZIP10, and bZIP25 and their equimolar mixtures

**Figure 2:**
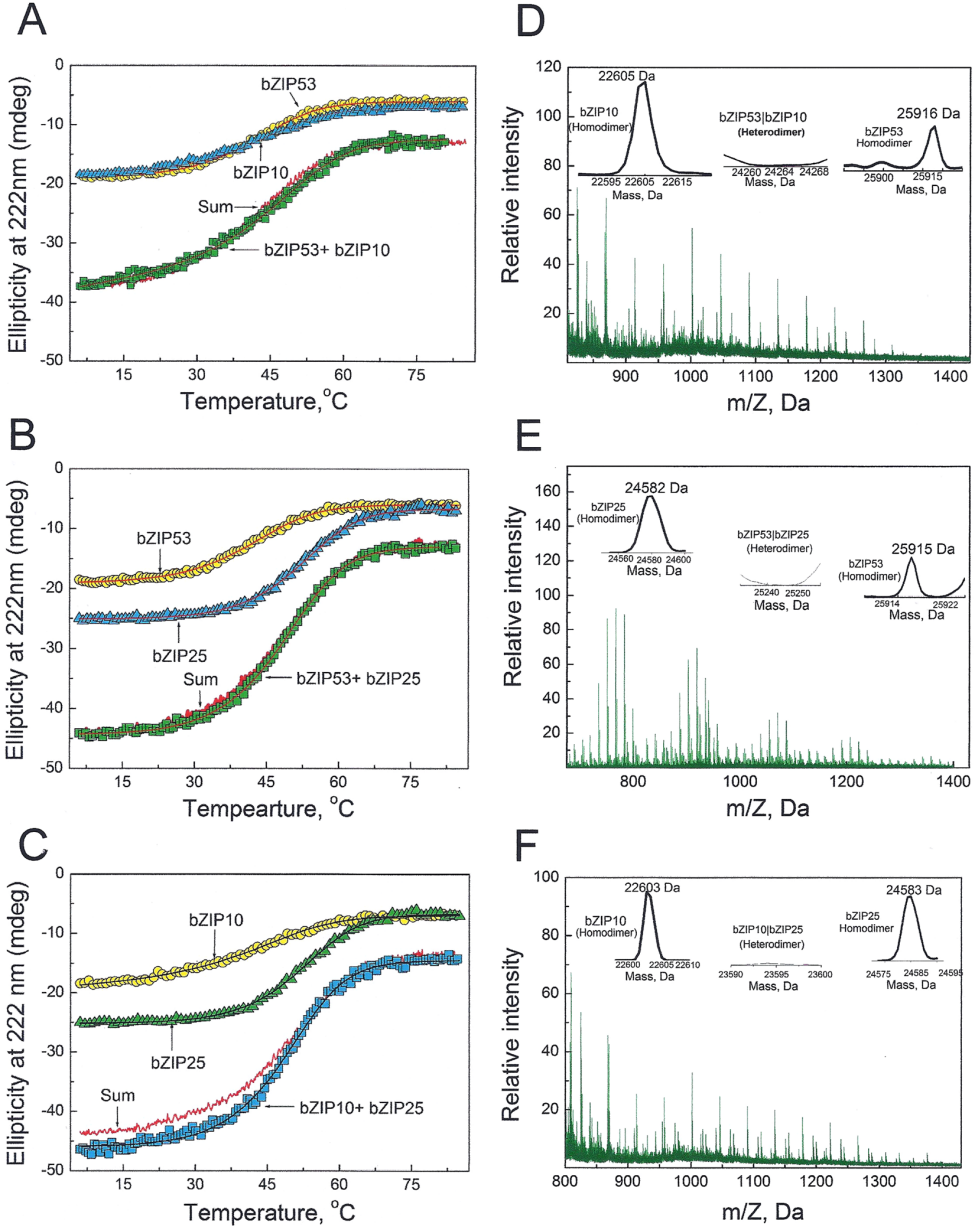
Representative CD thermal denaturation profiles and ESI-Mass spectrum of 2 µM (dimer) each of bZIP53, bZIP10, and bZIP25, and their equimolar mixtures (4 µM dimer). (**A**) Thermal denaturation curves of bZIP53, bZIP10, and their equimolar mixture were monitored at 222 nm (indicative of α-helices) when buffered protein solutions were heated. Both proteins were 2 µM therefore mixtures were 4 µM. bZIPs helical contents decreased as the temperature was increased from 6–85 °C. The sum line (smooth curve) represents the theoretical sum of two protein profile assuming no interaction between proteins. (**B**) Thermal denaturation of bZIP53, bZIP25, and their equimolar mixture. (**C**) Thermal denaturation of bZIP10, bZIP25, and their equimolar mixture. (**D**) ESI-Mass spectra of mix of bZIP53, and bZIP10 (each 2 µM dimer). (**E**) ESI-Mass spectra of mixture bZIP53 and bZIP25 (each 2 µM dimer). (**F**) ESI-mass spectra of mix of bZIP10, and bZIP25 (each 2 µM dimer).

To understand if the three bZIP proteins used in this study form heterodimers in solution, we used CD spectroscopy at 222 nm to monitor the thermal stability of bZIP53, bZIP10, and bZIP25, and their equimolar mixtures (Fig. 2A–C). It is assumed that a heterodimer between two proteins in a mixture will only be formed if heterodimer is more stable than either of the homodimers. Figure 2A shows the temperature-induced melting curves of bZIP53 (2 µM dimer), bZIP10 (2 µM dimer), and their mixture (4 µM dimer). Both proteins show cooperative unfolding as the temperature was increased from 6–85 °C. Assuming thermal denaturation of leucine zipper domains to be of two-state type, each melting curve was fitted according to Equation 1 (supplementary methods) that gave values of T_m_ and ΔH_m_. These along with ΔC_p_ of −1.79 kcal mol^−1^ dimer^−1^ K^−1^ (Supplementary Figure S1) were used to obtain ΔG_Di_ at 25 °C using Equation 2 (supplementary methods). Dissociation constant (K_D_) at 25 ^o^C was calculated using the expression ΔG_Di_ = −**R**TlnK_D_. All such thermodynamic stability parameters are given in Table 1. Also shown in Fig. 2A is the theoretical sum line of the mixture if two proteins do not interact. Thermal denaturation curve of mixture closely follows the sum line curve suggesting that these two proteins do not form a heterodimer. In another experiment, bZIP53 was mixed with bZIP25 and heated from 6–85 °C. Figure 2B shows the CD thermal denaturation curves of bZIP53 and bZIP25 alone and their mixture. bZIP25 melts with a symmetrical thermal profile and is well-fitted according to Equation 1. Thermodynamic parameters associated with thermal denaturation of bZIP25 are given in Table 1. bZIP25 is more stable and shows higher T_m_ and ΔG_Di_ values compared to bZIP53 and bZIP10. Like bZIP10, bZIP53 did not interact with bZIP25 shown by the near overlap of mixture and sum line curve. Thermal denaturation profiles of bZIP10 and bZIP25 and their mixture are shown in Fig. 2C. Calculated sum curve and experimentally obtained mixture curve (bZIP10 + bZIP25) were similar, suggesting that these proteins did not heterodimerize.

**Table 1:** Stability parameters associated with thermal denaturation of three bZIP proteins and their equimolar mixtures.

Protein	Homodimers			Heterodimers			
	T_m_^a^ [°C]	ΔH_m_^b^ [kcal mol^−1^ dimer^−1^]	ΔG_Di_^c^ [kcal mol^−1^ dimer^−1^] (25 °C)	Protein mixture	T_m_ [°C]	ΔH_m_ [kcal mol^−1^ dimer^−1^]	ΔG_Di_ [kcal mol^−1^ dimer^−1^] (25 °c)
bZIP53	41.9	−35.5 ± 4	−10.0 ± 0.2	bZIP53 + bZIP25	^d^ND		
bZIP25	52.2	−55.3 ± 5	−14.0 ± 0.4	bZIP53 + bZIP10	ND		
bZIP10	47.5	−41.1 ± 6	−11.6 ± 0.4	bZIP25 + bZIP10	ND		

^a^T_m_ is the midpoint of thermal denaturation. Error in the T_m_ values of three independent measurements was ≤0.5 °C. ^b^ΔH_m_ is the enthalpy change at T_m_. Values are the mean of three independent measurements, and represents ± standard error. ^c^ΔG_Di_, the free energy change of dimer formation at 25 °C were calculated using the values of ∆H_m_ at corresponding T_m_ and an experimentally obtained ΔC_p_ value of −1.74 ± 0.13 kcal mol^−1^ dimer^−1^ K^−1^. ^d^Since proteins did not interact, stability parameters were not determined (ND).

### Mass spectrometry revealed the oligomer states of bZIP53 and its interaction with bZIP10, and bZIP25

Mass spectrometry was used to study oligomer states of proteins. Electrospray ionization-mass spectrometry (ESI-MS) chromatographs showed *m*/Z charge series for bZIP53 proteins (Supplementary Figure S2A,B). *m*/Z signals were converted to molecular mass peaks using Bioanalyst software. bZIP53 protein exist predominately as monomer and dimer (bZIP53_monomer_ = 12957 Da, bZIP53_dimer_ = 25914 Da). Furthermore, we used mass spectrometry to characterize dimeric complexes in bZIP53 and bZIP10 protein mix. Figure 2D shows the m/Z chromatograph of mixture of 2 µm each of bZIP53 and bZIP10. The chromatograph was analyzed for molecular mass species in the mixture. We obtained two peaks at 22605 and 25916 Da that corresponds to homodimeric bZIP10 and bZIP25, respectively. Absence of molecular mass peak in and around 24260 Da means that bZIP53 and bZIP10 do not heterodimerize. Figure 2E show the ESI-MS spectra of mixture of 2 µM each of bZIP53 and bZIP25. Molecular mass peaks that correspond to bZIP25 homodimer (24582 Da), and bZIP53 homodimer (25916 Da) without a heterodimer peak were observed. Figure 2F show mass spectrum of equimolar mix of bZIP10 and bZIP25. Peaks at 22603 Da (bZIP10 dimer) and 24583 Da (bZIP25 dimer) were observed. Spectrum is characterized by the absence of any heterodimeric peak (~23593 Da) indicating absence of any interaction between bZIP10 and bZIP25. In all experiments, absence of heterodimer molecular mass peaks augments well with our CD thermal denaturation results both showing absence of interactions between three bZIPs.

### Electrophoretic Mobility Shift Assay (EMSA) and CD thermal denaturation experiments demonstrated that three bZIP proteins bind to G-box DNA and DNA binding increased bZIP53 α-helicity and thermal stability

**Figure 3:**
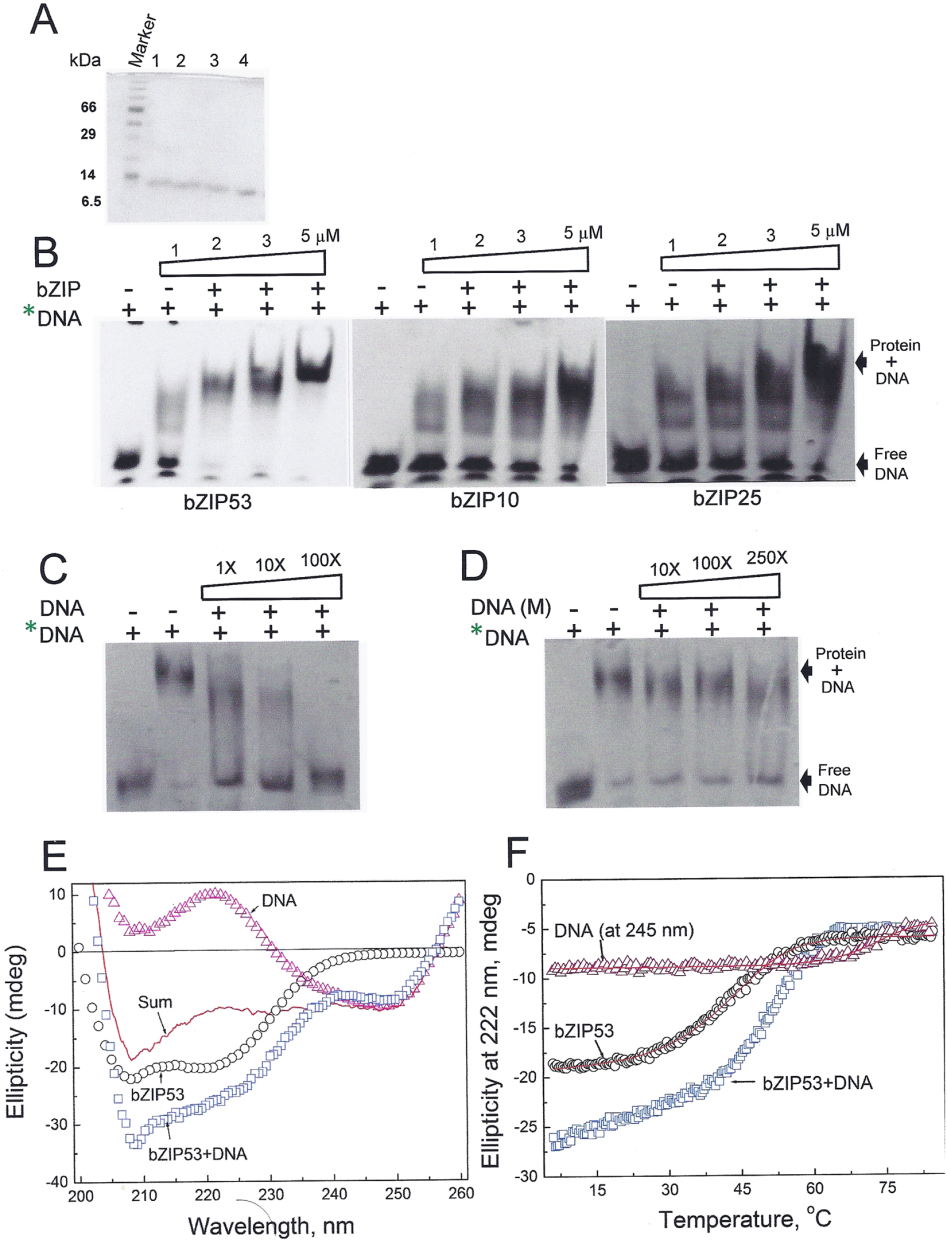
Binding of three bZIPs to a G-box containing DNA. (**A**) SDS-PAGE of 10 μg of 1: bZIP53, 2: bZIP25, 3: bZIP10 and 4: A-ZIP53. All proteins were homogenous and showed single band. (**B**) EMSA showing the dose-dependent binding of bZIP53, bZIP10 and bZIP25 to fluorescein end labelled 28 bp DNA containing a G-box sequence (ACGTG). 1, 2, 3, and 5 µM of bZIP53, bZIP10, and bZIP25 were incubated with 0.5 µM 28 bp fluorescein labelled double-stranded DNA (*DNA) in 20 µl reaction volume. 12 µl of reaction mix was loaded in 5% native PAGE and was run for 1 hour at 4 °C. Resolved gel showed the dose-dependent binding of all three proteins. (**C**) Competition assay using unlabeled G-box containing DNA (DNA lane). (**D**) Competition assay using mutated G-box (ACGTA) (DNA(M) lane). (**E**) bZIP53 become more helical and showed increased stability when bound to the DNA. CD wavelength scans of 2 µM bZIP53, 2 µM unlabeled 28 bp ds DNA containing a G-box for bZIP53 binding, and their mix. There is an increase in helicity of bZIP53 at 222 nm in the presence of DNA, indicative of unordered basic DNA binding region of bZIP53 becoming helical when bound to its cognitive DNA binding site. (**F**) Thermal denaturation of ds DNA monitored at 245 nm. Also shown is the CD melting curves of bZIP53 in the absence and presence of DNA.

EMSA or gel shift assays and CD experiments were used to show the DNA binding of bZIP53, bZIP10 and bZIP25 and effect of DNA binding on structure and stability of bZIP53. Figure 3A shows the SDS-PAGE run of HPLC purified bZIP53, bZIP25, bZIP10 and A-ZIP53. Single band for each preparations mean that proteins were homogenous. Gel shift experiments were performed with bZIP53, bZIP10, bZIP25, and 28 bps ds DNA containing a unique G-box sequence (ACGTG). One strand was fluorescein labelled at 5′-end. For each experiment 1 µM of labeled dsDNA was incubated with increasing concentrations of the indicated bZIP proteins. The mixtures were equilibrated for 1 hour and resolved on a 5% native PAGE. Upper shifted bands indicated DNA and protein complexes (Fig. 3B). These experiments showed our predictions that define N-and C-termini for these three bZIPs proteins are valid. For the experimental conditions used here, DNA bound bZIP complexes show low mobility and bands are super shifted with increasing concentrations of bZIPs. We interpret these results on the basis of specific and non-specific binding. Initially, bZIP binds specifically to G-box but at higher concentrations it binds non-specifically to the other parts of DNA probe. Similar observations were made in earlier studies^24,25^. Because of the similar size of proteins used in gel shift experiment we were unable to conclusively distinguish heterodimeric bands. To demonstrate that bZIP53 binds specifically to G-box, two sets of competition experiments were performed. In first experiment, G-box binding of bZIP53 was challenged by increasing concentrations of unlabeled probe. bZIP53 binding is completely abrogated at 100X of unlabeled probe (Fig. 3C). In second experiment even 250X of unlabeled probe with a mutation in G-box (ACGTA) did not affect the bZIP53 binding to consensus G-box (Fig. 3D).

CD studies were used to show changes in bZIP53 structure and stability upon DNA binding. Figure 3E shows the isothermal (6 °C) CD wavelength spectra of bZIP53 (2 µM), 28 bps DNA (2 µM) with a G-box and their equimolar mix. bZIP53 protein’s CD spectrum showed minima at 222 nm and 208 nm, a characteristic feature of α-helices. DNA showed minima at 245 nm. Their mix showed a clear increase in negative ellipticity at both 222 nm and 208 nm that indicated to unstructured basic region becoming α-helical upon DNA binding, as observed for other bZIP and b-HLH-ZIP TFs^26–28^. In order to probe if bZIP53 binding to DNA results in increased stability, we performed CD thermal denaturation studies. Melting studies of 28 bps DNA was carried out at 245 nm at which changes in ellipticity with increasing temperature were significant. Heat-induced changes in ellipticity signal of the mix of bZIP53 and DNA were observed at 222 nm at which DNA contribution to total optical signals were negligible and did not change with increasing temperature. bZIP53 thermal stability increased from 41.9 °C to 52.5 °C (Fig. 3F). The results suggested that increased secondary structure and thermal stability following DNA binding were concurrent.

### Designed dominant negative A-ZIP53 heterodimerized with bZIP53, bZIP25 and bZIP10 and prevented their DNA binding

**Figure 4:**
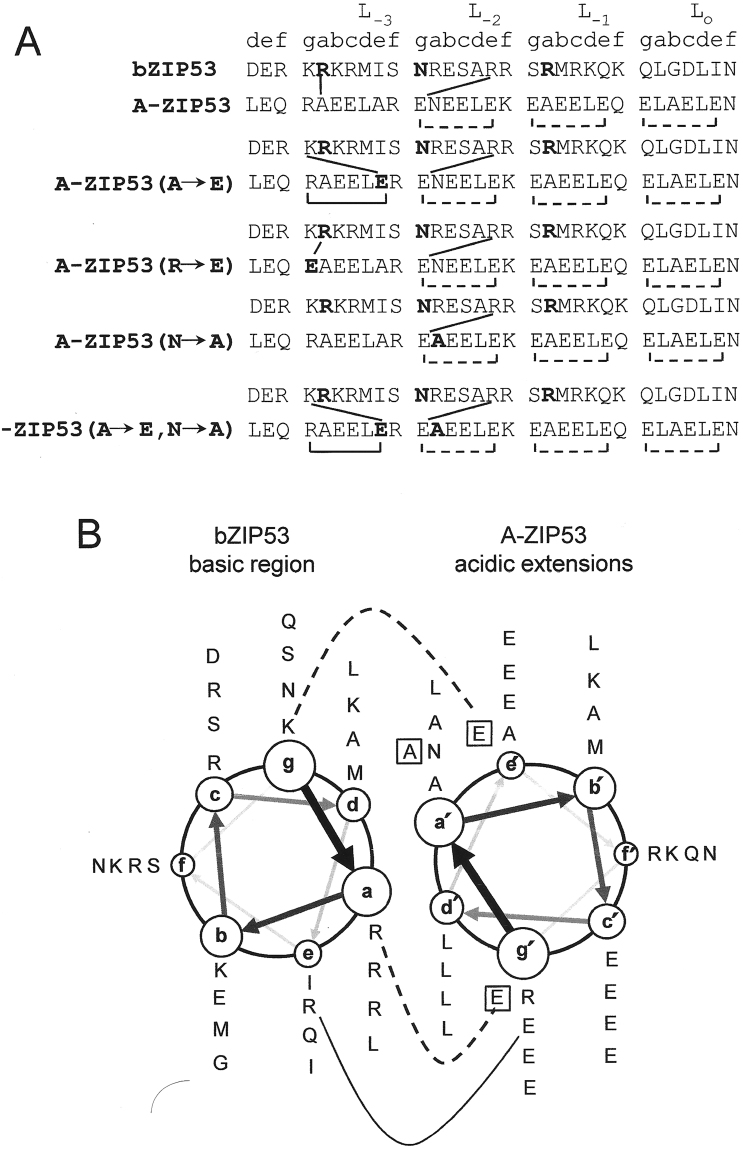
Heterodimers between A-ZIP53, its substitutes, and the wild-type bZIP53 are more stable than the homodimers. (**A**) Alignment of acidic helical extensions used in this study with DNA-binding region of wild-type bZIP53 showing the possible interhelical interactions in homodimers and heterodimers. At the top is shown the coiled coil heptad designations (*a,b,c,d,e,f*, and *g*). A-ZIP53 acidic extension, three single substitutes (A → E, N → A, and R → E) and a double substitute (A → E and N → A) of acidic extension are aligned with the basic region of bZIP53. Mutated amino acids, invariable asparagine at *g* position (L_-2_), and arginine at a position (L_-3_) are shown in bold. In homodimer coiled coil, interhelical interactions between amino acids in the *g* position with those in the following *e*’ position are shown as square brackets. Solid square brackets depict attractive interactions between oppositely charged amino acids in *g* and *e*’ positions whereas broken square brackets shows repulsive interactions due to the presence of similar charges (E ↔ E). In the putative heterodimeric coiled-coil, attractive interactions between *g* and *e*’ positions amino acids are shown by solid diagonal lines. (**B**) Coiled coil helical wheel diagram of the interactions in **A**, looking from the N-terminus. The coiled coil sequence reads outward from N- to C-termini around the wheel, starting at the *g* position of L_-3_. Possible electrostatic interactions between *g* ↔ *e*’ and *g* ↔ *a*’ are shown. Amino acids in the square brackets depicted the changes in the acidic extension of different A-ZIP53 versions used in this study. Solid line represents the interaction between A-ZIP53 with the wild-type bZIP53 and segmented line connects the mutants with the wild-type bZIP53.

**Figure 5:**
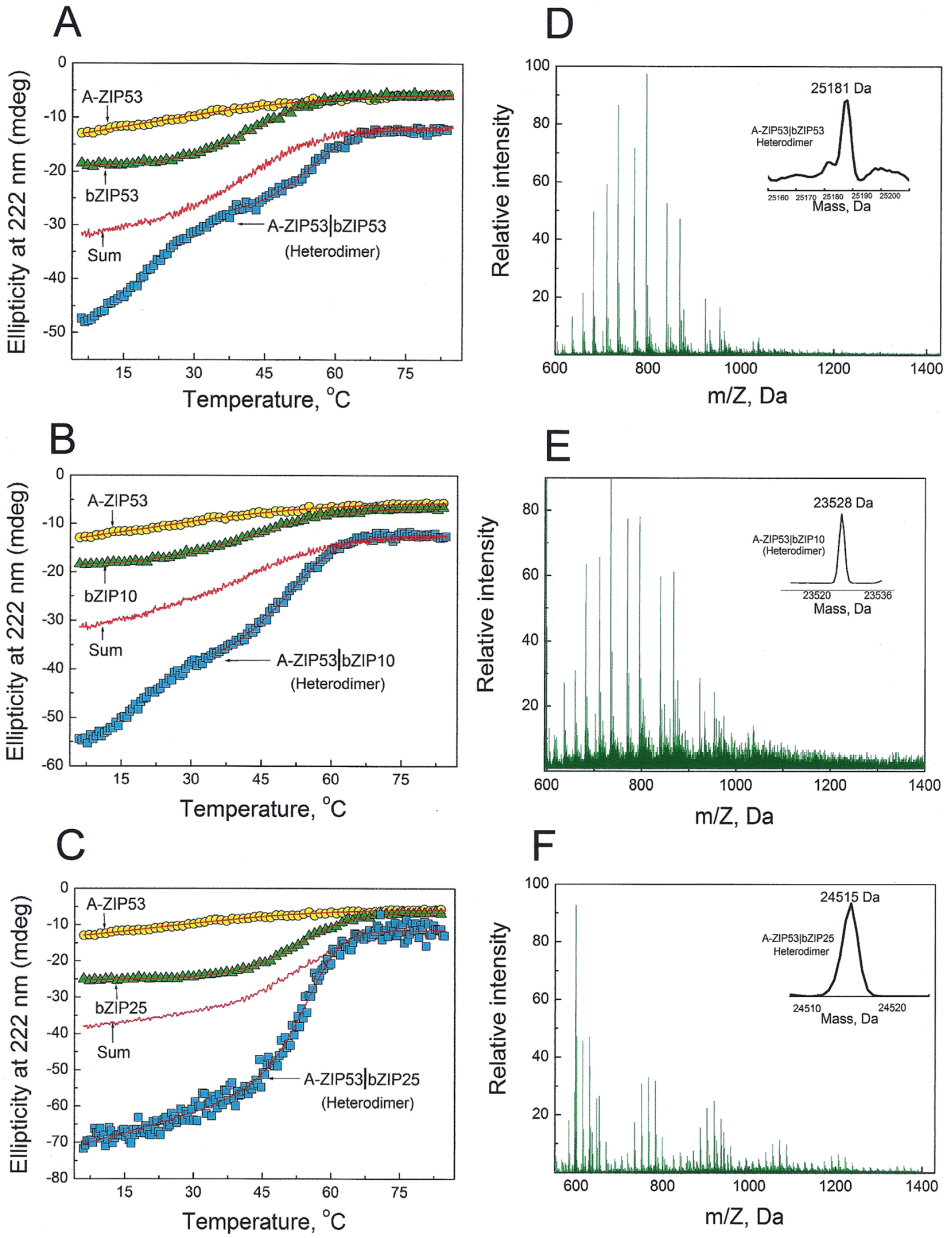
Representative CD thermal denaturation profiles and ESI-Mass spectrum of bZIP53, A-ZIP53, bZIP10, and bZIP25 (each 2 µM dimer), and their equimolar mixtures (4 µM dimer). (**A**) Thermal denaturation curves of bZIP53, A-ZIP53, and their equimolar mixture were monitored at 222 nm when buffered protein solutions were heated. bZIPs helical contents decreased as the temperature was increased from 6–85 °C. The sum line (smooth curve) represents the theoretical sum of two protein profiles assuming no interaction between these proteins. (**B**) Thermal denaturation of bZIP10, A-ZIP53, and their equimolar mixture. (**C**) Thermal denaturation of bZIP25, A-ZIP53, and their equimolar mixture. (**D**) ESI-Mass spectra of mix of bZIP53, and A-ZIP53 (each 2 µM dimer). (**E**) ESI-Mass spectra of mixture bZIP10, and A-ZIP53 (each 2 µM dimer). (**F**) ESI-Mass spectra of mix of bZIP25, and A-ZIP53 (each 2 µM dimer).

To address the query, if bZIP53, bZIP10 and bZIP25 can heterodimerize in the presence of DNA, we designed a dominant negative version of bZIP53 termed A-ZIP53. A-ZIP53 contains the bZIP53 leucine zipper but the DNA binding basic region is replaced with an amphipathic acidic peptide sequence, rich in glutamic acid that acts as a DNA mimetic and also forms a heterodimeric coiled coil with the basic region^29^. Oligomer state of A-ZIP53 was probed using ESI-MS. Supplementary Figure S2C,D shows m/Z chromatograph of 2 μM A-ZIP53 protein. m/Z signals were converted to molecular mass peaks using Bioanalyst software. A-ZIP53 showed major peaks at 12221 and 24442 Da that correspond to its monomer and dimer conformations. Figure 4A,B and Supplementary Figure S3A,B show the alignment of amino acids sequences of basic DNA binding region of bZIP53, bZIP10, and bZIP25 with designed acidic sequence. CD monitored heat-induced denaturations of bZIP53, the A-ZIP53, and their equimolar mixture are shown in Fig. 5. A-ZIP53 is unstable under the experimental conditions but had a well-defined post-transition baseline that allowed us to fit the transition curve according to Equation 1 using the pre-transition parameters of A-ZIP53 (A → E), a derivative of A-ZIP53 that will be described in the following write-up. The experimental curve of the mixture of A-ZIP53 and bZIP53 did not match the theoretical sum line curve indicating that these proteins interact. A-ZIP53|bZIP53, with a vertical bar represents a stable heterodimer between A-ZIP53 and bZIP53 (Fig. 5A). Thermal denaturation profile of A-ZIP53|bZIP53 show two phases. We interpret low temperature melt (6–35 °C) as fraying of N-terminal end of A-ZIP53|bZIP53 heterodimer. Only high temperature denaturation curve that represents leucine zipper melting was used for fitting. Thermodynamic stability parameters (T_m_, ΔH_m_, and ΔG_Di_) obtained for A-ZIP53|bZIP53 melting curve are given in Table 2. Based on K_D_ value A-ZIP53|bZIP53 is approximately three orders of magnitude more stable than the bZIP53 homodimer.

**Table 2:** Stability parameters associated with thermal denaturation of three bZIP proteins and their mixtures with designed dominant negative proteins.

Protein	Homodimers			Heterodimers			
	T_m_^a^ [°C]	ΔH_m_^b^ [kcal mol^−1^ dimer^−1^]	ΔG_Di_^c^, (K_D_)^d^ [kcal mol^−1^ [M] dimer^−1^]	Protein mixture	T_m_ [^o^C]	ΔH_m_ [kcal mol^−1^dimer^−1^]	ΔG_Di_, (K_D_) [kcal mol^−1^ [M] dimer^−1^]
bZIP53	41.9	−35.5 ± 4	−10.0 ± 0.2 (5e-8)	bZIP53 + A-ZIP53	51.5	−64.9 ± 2	−14.6 ± 0.2 (2e-11)
bZIP25	52.2	−55.3 ± 5	−14.0 ± 0.4 (5e-11)	bZIP53 + A-ZIP53 (A → E)	52.3	−54.3 ± 5	−13.9 ± 0.4 (6e-11)
bZIP10	47.5	−41.1 ± 6	−11.6 ± 0.4 (3e-9)	bZIP53 + A-ZIP53 (N → A)	53.9	−69.3 ± 2	−15.7 ± 0.2 (3e-12)
bZIP39	46.8	−43.2 ± 5	−11.6 ± 0.3	bZIP53 + A-ZIP53 (R → E)	53.1	−57.4 ± 3	−14.4 ± 0.2 (3e-11)
bZIP72	55.1	−57.3 ± 4	−15.0 ± 0.4	bZIP53 + A-ZIP53 (A → E, N → A)	55.7	−67.8 ± 4	−16.4 ± 0.4 (9e-13)
A-ZIP53		unstable		bZIP25 + A-ZIP53	54.2	−66.6 ± 5	−15.6 ± 0.5 (4e-11)
A-ZIP53 (A → E)	25.5	22.0 ± 7	−7.4 ± 0.1	bZIP25 + A-ZIP53 (A → E)	55.4	−75.0 ± 6	−16.8 ± 0.5 (5e-13)
A-ZIP53 (N → A)		unstable		bZIP25 + A-ZIP53 (N → A)	55.8	−63.3 ± 2	−15.8 ± 0.3 (3e-12)
A-ZIP53 (R → E)		unstable		bZIP25 + A-ZIP53 (R → E)	54.7	−62.9 ± 3	−15.4 ± 0.4 (5e-12)
A-ZIP53(A → E, N → A)		unstable		bZIP25 + A-ZIP53 (A → E, N → A)	57.6	−71.2 ± 4	−17.2 ± 0.4 (2e-13)
				bZIP10 + A-ZIP53	50.8	−56.9 ± 5	−13.7 ± 0.4 (9e-11)
				bZIP10 + A-ZIP53 (A → E)	51.1	−50.9 ± 5	−13.3 ± 0.4 (2e-10)
				bZIP10 + A-ZIP53 (N → A)	53.3	−65.5 ± 5	−15.2 ± 0.4 (7e-12)
				bZIP10 + A-ZIP53 (R → E)	50.9	−58.7 ± 6	−13.9 ± 0.5 (6e-11)
				bZIP10 + A-ZIP53 (A → E, N → A)	55.5	−60.8 ± 3	−15.5 ± 0.3 (4e-12)
				bZIP39 + A-ZIP53	^d^ND		
				bZIP72 + A-ZIP53	ND		

^a^T_m_ is the midpoint of thermal denaturation. Error in the T_m_ values of three independent measurements was ≤ 0.5 °C. ^b^∆H_m_ is the enthalpy change at T_m_. Values are the mean of three independent measurements, and represents ± standard error. ^c^G_Di_ are the values of the free energy change of dimer formation at 25 °C and were calculated using the values of ∆H_m_ at corresponding T_m_ and a experimentally obtained ΔC_p_ value of −1.74 ± 0.13 kcal mol^−1^ dimer^−1^ K^−1^. ^d^K_D_ is the dissociation constant of dimerization at 25 °C. ^d^Since proteins did not interact stability parameters were not determined (ND).

Interactions of A-ZIP53 with bZIP10 (Fig. 5B) and bZIP25 (Fig. 5C) were tested by heating their equimolar mix. As in A-ZIP53|bZIP53, A-ZIP53|bZIP10 melted in two phases and high temperature profile that corresponds to leucine zipper melting was fitted according to Equation 1. Since both the mixture curves i.e., A-ZIP53|bZIP10 and A-ZIP53|bZIP25 did not superimpose and showed higher T_m_ than the respective sum curves, we interpret this as stable heterodimers. Figure 5D shows the ESI-MS spectra of mixture of 2 μM each of A-ZIP53 and bZIP53. Figure 5E,F present the A-ZIP53 + bZIP10 and A-ZIP53 + bZIP25 interactions. m/Z spectra were converted into the molecular mass spectra. Molecular mass peaks at 25181, 23527, and 24515 Da that correspond to heterodimeric A-ZIP53|bZIP53, A-ZIP53|bZIP10, and A-ZIP53|bZIP25 respectively, were observed (inlets Fig. 5D–F). Under the experimental conditions we did not detect any monomeric mass peaks.

### A-ZIP53 heterodimerized with bZIP53, bZIP10 and bZIP25 and inhibited their DNA binding

**Figure 6:**
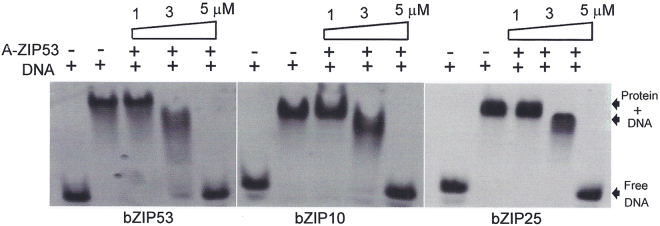
A-ZIP53 inhibits the DNA binding of three bZIPs. (**A**) EMSA showing the G-box DNA binding of all three bZIPs that were completely inhibited by equimolar concentration of A-ZIP53. 1, 3, and 5 µM of A-ZIP53 was heated with 5 µM each of bZIP53, bZIP10, and bZIP25 and incubated for 5 min at room temperature. 0.5 µM fluorescein labelled DNA was added and the mixtures were incubated for another 1 hour. DNA-Protein complexes were resolved on 5% native PAGE. Inhibition of DNA binding by A-ZIP53 showed dose-dependence with complete inhibition of binding at equimolar concentration.

EMSA was also used to show that A-ZIP53 inhibited the G-box DNA binding of bZIP53, bZIP10, and bZIP25. Figure 6 shows the results of a gel shift experiments after adding increasing concentrations of A-ZIP53 to a DNA binding reactions containing 5 μM bZIP53 (left panel), bZIP10 (middle panel), bZIP25 (right panel), and 1 μM 28 bps DNA with a G-box binding site. A-ZIP53 completely abolished DNA binding of bZIP53, bZIP10, and bZIP25 at equimolar concentrations. Similar to results in Fig. 3B, A-ZIP53 at lower concentrations affects the bZIP53, bZIP10 and bZIP25 binding to outside of G-box consensus sequence.

### A-ZIP53 specifically interacted with three bZIPs but did not dimerize with other bZIP proteins

**Figure 7:**
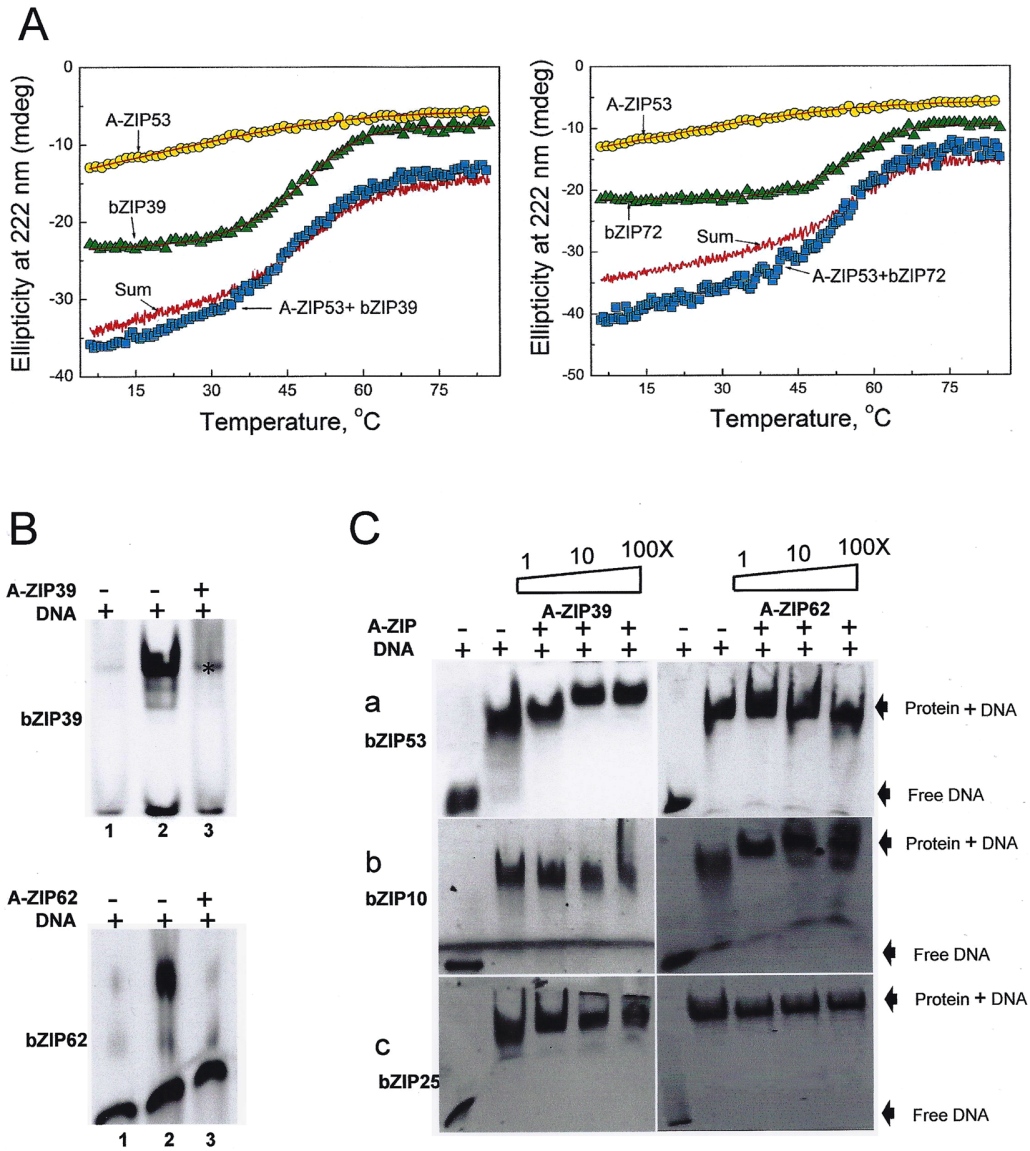
CD thermal denaturation studies and EMSA show the specificity of A-ZIP53. (**A**) Left panel: CD thermal denaturation curves of A-ZIP53 (2 µM dimer), bZIP39 (2 µM dimer), and their equimolar mix (4 µM dimer). Theoretical sum of thermal denaturation curve of A-ZIP53 and bZIP39 mix closely resembles experimental transition curve indicating no interaction between the two proteins. The results suggest that attractive interaction between acidic extension of A-ZIP53 and basic DNA-binding region of bZIP39 cannot overcome repulsion between their leucine zipper regions. Similar results were obtained when A-ZIP53 was mixed with the bZIP72 and heat denatured (right panel). (**B**) Upper panel: A-ZIP39, a designed DN of bZIP39 inhibited the G-box DNA binding of 1 μM bZIP39 in equimolar concentration (*nonspecific band). Lower panel: Equimolar A-ZIP62 inhibited the G-box binding of 2 μM bZIP62. (**C**) EMSA showcasing the 1 μM each of bZIP53, bZIP10, and bZIP25 bound to G-box. In the competition experiments 1, 10, and 100 molar excess of A-ZIP39 and A-ZIP62 were added to the reaction mix containing bZIP53 (upper panel), bZIP10 (middle panel), and bZIP25 (lower panel), heated to 55 °C and incubated for 5 minutes at room temperature and mixed with 0.5 µM fluorescein labelled DNA. Even 100 molar excess of A-ZIP39 and A-ZIP62 did not abolish the binding of bZIP53. The results show that the leucine zippers of three bZIPs dictate their dimerization potential.

Whether two bZIPs will heterodimerize depends on if they are co-expressed. Transcript levels of all bZIPs during seed development and maturation are given in Supplementary Figure S4. To address the dimerization specificity of A-ZIP53, we chose bZIP39 and bZIP72, two bZIP proteins that are upregulated during maturation phase of seed development in *Arabidopsis* ^30,31^. Two sets of experiments were performed. In the first, A-ZIP53 ability to dimerize with other bZIP proteins was examined by measuring thermal stability of mixtures. Figure 7A shows thermal denaturation curves of 2 μM bZIP39, 2 μM A-ZIP53, and their equimolar mixture. The shape of the mixture curve is similar to that of the sum line curve suggesting these two proteins do not interact. Similarly, when A-ZIP53 was mixed with bZIP72, two proteins melted with a profile that is akin to sum line curve indicating that the dimerization domains of these two bZIP proteins are sufficiently different from either of the three bZIPs (bZIP53, bZIP10, and bZIP25) to prevent interactions even if stabilized by the interaction of the acidic region from A-ZIP53 and the basic region of these proteins.

In a second set of experiments, gel shifts were used that involved A-ZIP39 and A-ZIP62, the dominant negative forms of bZIP39 and bZIP62, respectively. The design strategy used to produce A-ZIP53 was also adapted to create A-ZIP39 and A-ZIP62 (supplementary information). Whereas DNA binding activities of bZIP53, bZIP10, and bZIP25 were completely abolished by equimolar concentration of A-ZIP53, their DNA bindings were not affected even by 100X equivalent (500 μM) of either A-ZIP39 or A-ZIP62 (Fig. 7B). The results showed that dimerization specificity of bZIPs lies in the leucine zipper region.

### Derivatives of A-ZIP53 with different homodimeric and heterodimeric stabilities

We strive to design dominant negatives of bZIPs that function *in vivo* ^11,13,14,32^. For dominant negative to be effective as a transgene, it must unfold into monomers and heterodimerize with targeted wild type bZIPs. We designed a series of dominant negative proteins, each with different homodimeric stability. Figure 4 shows the amino acid sequences of four additional versions of A-ZIP53 that were cloned, expressed, and biophysically characterized. First derivative A-ZIP53 (A → E) has a glutamic acid instead of an alanine at the *e* position in L_-3_ heptad this resulted in an additional salt bridge (K ↔ E’) in A-ZIP53 (A → E)|bZIP53 heterodimeric coiled coil. Substitution also introduced a salt bridge (K ↔ E’) in A-ZIP53 (A → E) homodimer.

**Figure 8:**
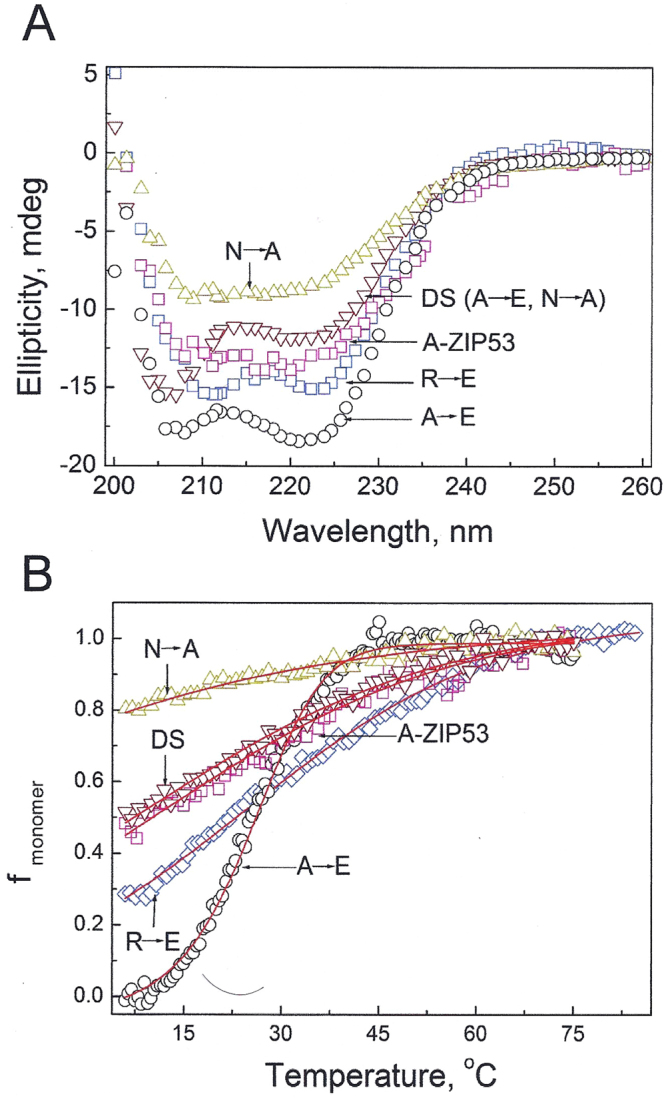
Designed DN proteins show different homodimer stabilities. (**A**) Isothermal (6 °C) CD wavelength scans of five dominant negatives used in this study. All proteins were at 2 µM dimer. DNs A-ZIP53, A-ZIP53 (N → A), A-ZIP53 (DS:N → A, R → E), and A-ZIP53 (R → E) gave CD signal ratio 222/208 < 1 suggesting the absence of stable coiled coil. However, A-ZIP53 (A → E) showed prominent peaks at 222 and 208 nm with 222/208 ratio > 1 suggesting the presence of stable coiled coil. (**B**) CD thermal denaturation of designed DN proteins. CD signals at 222 nm were converted into fraction monomer. A-ZIP53 (A → E) melted with symmetrical sigmoidal curve with well-defined dimeric protein baseline at lower temperature and was used for obtaining fraction monomer for other A-ZIP53 derivatives.

Derivative A-ZIP53 (R → E) introduced attractive *g ↔ a’* interaction (E ↔ R) in A-ZIP53 (A → E)|bZIP53 heterodimer. A-ZIP53 (N → A) mutant has an alanine instead of asparagine at *a* position of the L_-2_ heptad. N-N’ interaction at *a* position has the most negative coupling energy that strongly favors homodimerization^33^. Alanine instead of asparagine at *a* position should favors heterodimerization with bZIP53. A double substitution dominant negative (A → E, N → A) was also designed and expressed. Isothermal CD scans at 6 °C were conducted for five dominant negative proteins and are shown in Fig. 8. CD scan of A-ZIP53 (A → E) shows 222/208 > 1 suggesting the presence of stable coiled coil. For other dominant negative proteins 222/208 ratios were <1 that are inferred as unstable coiled coils. CD traces of heat-induced profile of A-ZIP53 (A → E) showed a symmetrical melting curve with defined pre- and post- transition regions. Thermal denaturation curve was normalized and fraction monomer curve (f_monomer_) was plotted against temperature (Fig. 8B). Value of pre-transition of A-ZIP53 (A → E) was used to construct f_monomer_ for other mutant proteins. Analysis of melting curve of A-ZIP53 (A → E) gave T_m_ = 25.5 °C and ΔG_Di_ = −7.4 ± 0.1 kcal mol^−1^ dimer^−1^.

### A-ZIP53 derivatives interacted with bZIP53, bZIP25 and bZIP10 with different heterodimeric stabilities

A-ZIP53 (A → E) was heat denatured alone and with equimolar bZIP53 (Supplementary Figure S5A). A-ZIP53 (A → E)|bZIP53 heterodimer was formed and melted with T_m_ = 52.3 °C. A-ZIP53 (A → E)|bZIP53 thermal denaturation curve shows well-defined pre- and post-transition baselines and was well fit according to Equation 1 (Supplementary data). Supplementary Figure S5B-C show thermal stability of A-ZIP53 (A → E)|bZIP10 and A-ZIP53 (A → E)|bZIP25 heterodimers. Both heterodimers showed increased thermal stability *vis á vis* individual homodimers. Each curve was analyzed for T_m_, ΔH_m_, and ΔG_Di_ and these values are given in Table 2. Supplementary Figure S5D-F shows the thermal stability of A-ZIP53 (R → E)|bZIP53, A-ZIP53 (R → E)|bZIP10, and A-ZIP53 (A → E)|bZIP25 heterodimers. Analysis of melting curves gave stability parameters that are included in Table 2. A-ZIP53 (N → A) interaction with bZIP53 was demonstrated by CD thermal studies (Supplementary Figure S6A). Equimolar mix of two proteins melted with single transition curve with higher T_m_. Supplementary Figure S6B,C demonstrate the thermal stability curves of A-ZIP53 (N → A)|bZIP10 and A-ZIP53 (N → A)|bZIP25, as expected heterodimers showed higher stability. Finally, Supplementary Figure S6D–F shows stability of A-ZIP53 (A → E, N → A) in complex with bZIP53, bZIP10, and bZIP25. This mutant formed the most stable heterodimer with three bZIPs (Table 1). All designed dominant negative interacted strongly with bZIP53, bZIP10, and bZIP25 with K_D_ values that made them 2–5 magnitude more stable than either of the homodimer (Table 2).

### A-ZIP53 inhibited the DNA binding of bZIP53, bZIP10 and bZIP25 in the transient transfection assays using *Arabidopsis* protoplasts

**Figure 9:**
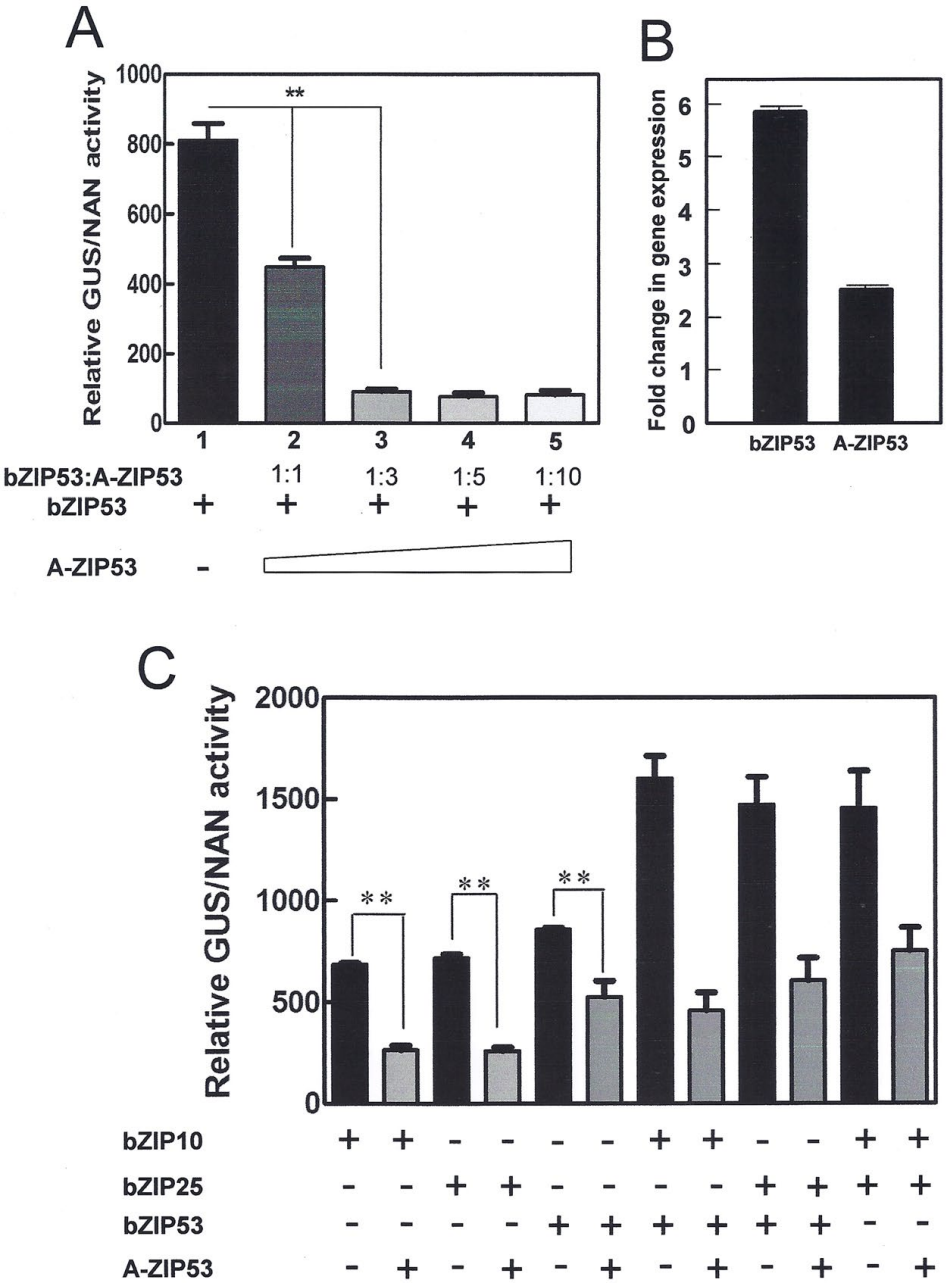
A-ZIP53 inhibited the bZIP53, bZIP10, BZIP25, and their heterodimers-mediated reporter gene activities in transient transfection experiments using *Arabidopsis* protoplasts. (**A**) Plasmid coding for bZIP53 can transactivate the *GUS* reporter gene under the control of the 2S2 promoter that contains a G-box binding site. ** represent P < 0.01. Protoplasts were co-transfected with plasmids coding for bZIP53 and A-ZIP53. Transient expressions of A-ZIP53 inhibited the bZIP53-mediated *GUS* reporter activity in dose-dependent manner. Column 2–5 depict reporter activity at bZIP53:A-ZIP53 molar ratio of 1:1, 1:3, 1:5, and 1:10, respectively. (**B**) qRT-PCR shows the transcripts of A-ZIP53 and bZIP53 in the transfected protoplasts (1:1 sample). (**C**) bZIP10 and bZIP25 overexpression can transactivate the *GUS* reporter gene, suggesting that these bZIPs can bind to the G-box containing promoter *in vivo*. Reporter activity was inhibited in the presence of 3 molar excess of A-ZIP53 plasmid suggesting that A-ZIP53 can compete with G-box for bZIP53 binding. Enhanced *GUS* activity was observed when protoplasts were co-transfected with 9 µg each of bZIP53, bZIP10, and bZIP25 in three possible combinations. A synergistic increase in reporter activities when two bZIP plasmids were co-transfected indicates to the formation of heterodimers. A-ZIP53 inhibited the reporter gene activity at 3 molar excess concentrations. Error bars represent standard deviation of three independent experiments.

Promoter analysis, ChIP assay, transient transfections, and EMSA confirmed the binding of bZIP53, bZIP10, and bZIP25 to the G-box present in the promoter of seed specific genes^18,34,35^. To probe the affectivity of A-ZIP53 against target bZIPs, transient transfection assays were performed in *Arabidopsis* protoplasts. For transfections, DNA construct of reporter plasmid (GUS expression under 2S2 promoter) was co-transfected with effector plasmids (A-ZIP53, bZIP53, bZIP10, and bZIP25) and NAN expressing control plasmid^18,23^. A-ZIP53 plasmid when co-transfected with bZIP53 plasmid decreased GUS signals in dose- dependent manner. It marked the inhibition of bZIP53 DNA binding by A-ZIP53 (Fig. 9A). Transformed protoplasts were checked for the presence of A-ZIP53 and bZIP53 transcript using qRT-PCR (Fig. 9B). Additionally, protoplasts were transiently transfected with construct of bZIP53, bZIP10, and bZIP25 in pairs as shown in Fig. 9C. The experiments resulted in significant increase in the normalized GUS reporter signals that indicated to the formation of putative heterodimers between three bZIPs *in vivo*. Gus reporter signals decreased substantially in presence of A-ZIP53. These results confirm our *in vitro* results and validated the use of A-ZIP53 *in vivo*.

## Discussion

Genome-wide studies predicted 67 and 72 bZIP transcription factors in *Arabidopsis* ^10,15^. Based on their dimerization properties, dictated by a strategically placed asparagine at *a* position and charged amino acids in *g* and *e* positions in each heptad of a leucine zipper, *Arabidopsis* bZIPs are classified as preferentially homdimerizing, homo- or heterodimerizing, and preferentially heterodimerizing^10^. The homodimerizing family has 14 groups (A-N) with 47 bZIP member proteins. bZIP10 and bZIP25 belongs to group G/C and bZIP53 was placed in group H/S^10,15^. bZIP53 has been shown to bind to the G-box containing 2S2 gene promoter of albumin gene. It also heterodimerizes with bZIP10 and bZIP25 and binds to the same G-box sequence^18^. Unlike human bZIPs, that are typically 4–6 heptads, *Arabidopsis* bZIP leucine zippers can be 8 or more heptads long. We used the thermal denaturation studies to obtain the stability parameters of bZIP53, bZIP10, bZIP25, and their three possible heterodimers. Except for bZIP53 with a T_m_ of 41.9 °C, all other bZIPs have T_m_ close to 50 °C, similar to what is observed for human and mouse bZIPs. bZIP72 was identified as a bZIP protein^15,31^ but did not fulfill the stringent criteria of bZIPs used by other group^10^. We have included bZIP72 in our study to show the specificity of our designed A-ZIP53.

Analyzing the amino acids sequences of leucine zipper regions of bZIP53, bZIP10 and bZIP25, we predicted that these proteins would preferentially homodimerize. We have based our prediction on earlier reported coupling energies of individual amino acid in *a* ↔ *a*’ and *g* ↔ *e*’ pairs^36^. A summary of attractive and repulsive *g* ↔ *e*’ interactions for bZIP53, bZIP10 and bZIP25 in homo-and heterodimeric conformations are included (Fig. 1). In three homo- and three heterodimeric conformations, the homodimer would be a preferred conformation. For example, based on *g* ↔ *e*’ interactions, a presumed heterodimer between bZIP53 and bZIP10 has 3 attractive and 1 repulsive interactions while bZIP53 and bZIP10 homodimers each have 4 attractive and 2 (bZIP53) and 0 (bZIP10) repulsive interactions. This, along with favorable N ↔ N’ interaction in *a* position of the 2^nd^ heptads, V ↔ V’ in *a* position of 3^rd^ heptads, and L ↔ L’ in *a* position of 7^th^ and 8^th^ heptads, would dissuade heterodimerization. Similarly, for other pairs absence of additional attractive interactions in leucine zipper and charge repulsion between basic DNA binding regions would preclude heterodimer formation between bZIP53, bZIP10 and bZIP25.

Surprisingly, *ex vivo* complementation studies and over expression studies suggested formation of heterodimer among group C and S bZIP members^17,18,23^. Previous studies have used Bimolecular fluorescence complementation (BiFC) and yeast two-hybrid system to show interactions between three bZIPs^21,22^. Though BiFC and yeast two-hybrid system are high-throughput and widely used, both have limitations, for example, the major drawback of BiFC is that the fluorescent protein halves are prone to self-assembly that may give false positive signals in co-transfections assays^37^. Also technique is not revealing if interactions are DNA-mediated. Similarly, yeast two-hybrid system is also prone to generate false positives^38^. In previous studies, using BiFC to study interaction between bZIP53 and bZIP10, two groups have shown contrasting results. Weltmeier *et al*.^22^, showed strong interaction between bZIP53 and bZIP10 whereas study from Llorca CM. *et al*.^21^ proved otherwise. In order to probe the role of DNA in defining the dimerization specificity of three bZIP proteins used here, we produced A-ZIP53, a designed dominant negative. The design strategy adapted the idea that acidic extension would electrostatically mimic DNA and would offer an alternate binding for bZIP53 to interact^26^. The basic region of bZIP53, instead of binding in the major groove of DNA would interact with acidic extension in A-ZIP53. This acidic adjunct extends the coiled coil structure from the leucine zipper region to the basic region in the A-ZIP53|bZIP53 heterodimer. Use of A-ZIP protein to understand the dimerization specificities among bZIPs is justified since *in vivo* dimerization is often driven by DNA binding.

Previously it was reported that A-ZIP protein drives interactions between weakly attractive or even somewhat repulsive leucine zippers^9^. As discussed above, bZIP53, bZIP10, and bZIP25 are predicted to form homodimers (Fig. 1) but this approach does not take into account the role of DNA in dimerization. Use of A-ZIP proteins that mimic DNA gives us access to measuring a range of bzip dimerization affinities *in vitro*. Use of A-ZIP proteins that mimic DNA gives us access to measuring a range of bZIP dimerization affinities *in vitro*. CD thermal denaturation studies of the mixture of A-ZIP53 and bZIP53 proteins show that A-ZIP53|bZIP53 formed a complex that melts with higher T_m_ than either of the two proteins (Fig. 5). Compared to the bZIP53 homodimer, the interaction stabilized the A-ZIP53|bZIP53 complex by 4.6 kcal mol^−1^ dimer^−1^ (Table 2). When A-ZIP53 was mixed in equal concentration with bZIP10 or bZIP25 and heat denatured, CD thermal denaturation profiles of A-ZIP53|bZIP10 and A-ZIP53|bZIP25 indicated the formation of stable heterodimers that were 2.1 and 2.5 kcal mol^−1^ dimer^−1^ more stable than homodimer bZIP10 and bZIP25.

ESI-MS molecular mass chromatogram of A-ZIP53 in complex with bZIP53, bZIP10, and bZIP25 showed prominent heterodimeric peaks reinforcing the fact these proteins are in stable complex. Similar results were obtained using EMSA. A-ZIP53 inhibited the DNA binding of bZIP53, bZIP10, and bZIP25 in equimolar concentrations. The ability of A-ZIP53 to specifically interact with group C and S bZIPs was demonstrated by thermal denaturation and EMSA experiments. A-ZIP53 failed to interact with bZIP39, a group A member, and bZIP72. Both are upregulated during seed formation in *Arabidopsis* ^30,31^. Furthermore, in EMSA experiments, two additional dominant negative proteins A-ZIP39 and A-ZIP67 failed to inhibit the DNA binding of bZIP53 even when present at 100 molar excess concentrations. Since the acidic extension interacts with all basic regions, the specificity of interaction between A-ZIP and bZIP domains lies in the leucine zipper region.

Using *Arabidopsis* protoplast, a system akin to animal cell lines, we checked if A-ZIP53 was active in a biological context. In a series of experiments, protoplasts were transfected with indicated combinations of bZIPs along with A-ZIP53 coding plasmids (Fig. 9). A-ZIP53 diminished the bZIP53 mediated GUS reporter activity. Experiments involving co-transfected bZIPs showed enhanced GUS signals compared to single plasmid. This is interpreted as enhanced gene expression due to formation of heterodimers^18,23^. A-ZIP53 inhibited bZIP protein mediated GUS activity in all experimental conditions used in this study. In conclusion, bZIP53, bZIP10, and bZIP25 can heterodimerize and dimerization is DNA-mediated and A-ZIP53 can compete with DNA for bZIP binding.

An ultimate aim of this study is to design dominant negative proteins that can be used to address the bZIP TF’s functions *in vivo*. Previous studies showed the specificity of A-ZIPs in biological system. For example A-C/EBP (a dominant negative of C/EBP family of bZIPs) when expressed as transgene in the mouse skin, regress pre-formed papilloma^13^ whereas expression of A-Fos, a dominant negative that inhibits the functions of Fos and Jun family of bZIPs, transdifferentiate papilloma into benign sebaceous adenomas^11^. These finding suggested that A-ZIPs are specific and different A-ZIPs target different signal transduction pathways. Furthermore, since A-ZIPs target family of closely related bZIPs they can be used to study functional redundancy, a phenomena common in biological systems^18^. Due to their simple design A-ZIP proteins may show off-target affect and each designed A-ZIP needs to be validated *in vitro* and *in vivo*. These designed dominant negatives may be effective molecular biology tools to understand the role of bZIP53 and its partners in seed formation and maturation. We have produced an A-ZIP expressing transgenic *Arabidopsis* and its molecular characterization is underway.

## Methods

### Plant material

*Arabidopsis thaliana* (ecotype Columbia 0) seeds were surface sterilized with 1% sodium hypochlorite, washed three times with sterile distil water, kept in dark at 4 °C for 2–3 days and grown on MS-agar plates. Germination was encouraged under controlled conditions and plants were grown at 22 °C, with 16 hours of light (150 µmol m^−2^ s^−1^) followed by 8 hours dark period cycles, at 60% relative humidity.

### RNA extraction and cDNA preparation for prokaryotic expression vectors

Total RNA was isolated from whole plant extract that included stem, leaves, flowers, and siliques using Spectrum™ Plant Total RNA Kit (Sigma-Aldrich, USA) and cDNA was prepared using Superscript® III Reverse Transcriptase kit (Invitrogen, USA) following manufacturer’s instructions. Full-length protein sequences with underlined cloned bZIP domains are given in supplementary information. Boundaries of bZIP domains were defined by the consensus amino acids in basic and leucine zipper regions^10,39^. Plasmid constructs of DNA binding domain and leucine zipper region of bZIP53 (Y^18^-D^116^), bZIP10 (V^216^-S^301^), bZIP25 (V^230^-S^322^), bZIP39 (V^351^-L^442^), bZIP62 (E^156^-S^251^), and bZIP72 (D^48^-T^150^) were prepared by PCR amplification of *Arabidopsis* cDNA and cloned into pT5 prokaryotic expression vector as BamHI-HindIII fragment^28^. Stop codon was introduced just before HindIII restriction site in cases where leucine zipper did not end naturally at C-terminus. Primers used for cloning of all bZIPs and dominant neagtive A-ZIPs used in this study are given in Supplementary Table S1. Amplified PCR product of bZIP53 contained an internal HindIII restriction site that was destroyed by site-directed mutagenesis and amplicon was cloned as BamHI-HindIII fragment. We were not able to amplify bZIP72 gene from the cDNA, therefore, it was synthesized using overlapping primers (Supplementary Table S1). For cloning dominant negative A-ZIPs, leucine zipper region of bZIP53, bZIP39, and bZIP62 were PCR amplified with forward primers containing XhoI site immediately upstream of L_o_ and reverse primers with BamHI site using respective bZIP plasmid as template. PCR products were digested and cloned as XhoI-HindIII in 4h-C/EBP plasmid^26^. These plasmids are named dominant negative A-ZIP53, A-ZIP39, and A-ZIP62. Derivatives of A-ZIP53 i.e., A-ZIP53 (A → E), A-ZIP53 (N → A), A-ZIP53 (R → E), and A-ZIP53 (A → E, N → A) were prepared by site-directed mutagenesis using combinations of primers given in Supplementary Table S1. Sequences of bZIP and A-ZIP proteins are given in supplementary information. All prokaryotic expression vectors were sequenced by dideoxy method using sequencing primers (Supplementary Table S1) and expressed protein sequences were confirmed by mass spectrometry and are included in supplementary information.

### Eukaryotic expression vectors

For cloning A-ZIP53 in the plant specific vector, pRI101 AN plasmid was double digested with NdeI-EcoRI. Using pT5 A-ZIP53 plasmid as template and forward and reverse primer with NdeI and EcoRI sites, 30 cycles of PCR were performed to amplify fragment containing T7 peptide sequence followed by A-ZIP53 domain. Amplicon was cloned into recipient pRI101 AN vector as NdeI-EcoRI double digested fragment following standard molecular biology protocols. Plasmids overexpressing full-length bZIP53, bZIP10, and bZIP25 under CaMV35S promoters, GUS reporter gene under 2S2 promoter, and NAN expressing plasmid under CaMV35S promoter were gifts from Prof. Wolfgang DrÖge-Laser, University of Würzburg, Germany. NAN coding plasmid was used for normalizing GUS reporter signals.

### Expression and purification of proteins

All proteins used here contain N-terminus 13 amino acids T7 tag (ASMTGGQQMGRDP). bZIPs and A-ZIPs expressing plasmids were transformed into *E. coli* BL21 (DE-3) strain following standard protocols^28^. 15% SDS-PAGE was used to demonstrate the homogeneity and purity of expressed proteins. 10 μg of bZIP53, bZIP10, bZIP25 and A-ZIP53 were subjected to electrophoresis and gel was stained with Coomassie blue. Sigma’s M6539 was used as molecular weight marker. Detailed methods are given in supplementary information.

### CD spectroscopy

CD isothermal studies were carried out at 6 °C to deduce the structural properties of bZIPs, A-ZIPs and G-box containing ds DNA. Thermal denaturation studies that measure the thermodynamic stability of homo-and heterodimeric proteins as a function of temperature were carried out using 815 spectropolarimeter (Jasco, Japan). Detailed methods are included in supplementary information.

### Protein sequencing by mass spectrometry

Amino acids sequences of expressed proteins were confirmed using Nano-LC MS/MS. Protein sequencing was performed on Triple TOF^M^ 5600 mass spectrometer (AB Sciex Pte. Ltd., USA) attached to the Nano-LC (Eksigent Technologies Llc, USA). For amino acids sequencing lyophilized tryptic digests of 0.1 μg of protein were resuspended in 0.1% formic acid^40^. 10 μl of sample was passed through desalting trap column (Chrom-XP, C-18-CL-3 μM, 350 μM × 0.5 mm, Eksigent Technologies LLC., USA). After desalting peptides were separated on C_18_ matrix (3C-18-CL-120, 3 μM, 120 A, 0.075 × 150 mm, Eksigent Technologies LLC., USA) before sequencing. The analyte was eluted using a gradient of mobile phase A (MS grade water with 0.1% formic acid) and mobile phase B (acetonitrile with 0.1% formic acid) from 5–95% mobile phase B in 32 min with a flow rate of 300 nl/min. After separation, peptides were subject to tandem mass (MS/MS) analysis. Peptides were identified using Protein Pilot software (Version 4.08085, AB Sciex Pte. Ltd., USA).

### EMSA experiments

5′- prime fluorescein labeled 28-mer HPLC purified oligonucleotides with single G-box (GTCAGTCAGGCCACGTGGCATGCCTCAG) consensus binding site for bZIP53, bZIP10, and bZIP25 was purchased from Sigma, USA. Same oligo was used for bZIP39 and bZIP62 binding studies. Molar excess of unlabeled complementary strand was mixed with fluorescein labeled oligo in TE buffer, heated to 65 °C and snap cooled on ice for 10 min in dark. Finally, 28 bp oligo was further incubated at room temperature for 30 min. Both homo-and heterodimer protein samples (0.5, 1, 3, and 5 µM dimer) were mixed in the gel shift buffer (10 mM Tris (pH 8.0), 150 mM KCl, 0.5 mM EDTA, 0.3% glycerol, 0.1% BSA, and 0.1% Triton-X 100) heated to 50 °C for five minutes and allowed to cool at room temperature. These samples were mixed with 0.5 µM double stranded (ds) oligo and incubated at room temperature for 1 hour. Reaction mixtures were loaded onto 5% native PAGE in 0.25X TBE buffer and resolved by applying 11 mA current (100 V) for 30 minutes at 4 °C. Prior to loading, gel was pre-run at 100 V for 15 min. Gel images were taken by measuring florescence signals using UVP BioSpectrum gel documentation system (excitation at 488, emission at 535 nm). For competition assay unlabeled consensus G-box (ACGTG) and mutant sequence (ACGTA) containing DNA was used.

### ESI-MS measurements

Protein solutions for ESI-MS were prepared in unbuffered MS grade water following method, essentially similar to earlier published protocol with some modifications^41^. All proteins were 2 μM homodimer and 4 μM heterodimer. If required pH of the solution was adjusted with the acetic acid or aqueous ammonia. ESI-MS measurements were performed on the Triple TOF^M^ 5600 mass spectrometer (AB Sciex Pte. Ltd., USA). For locking the bZIP samples in dimeric form, ion spray interface temperature was switched-off for all experiments and the counter-current gas flow was kept at 10. Declustering potential^42^ was set at 10 V. Prior to injection all samples along with the injection needle were kept at 23 ± 2 °C for at least 2 hours. Protein solutions were injected via capillary directly into the ionizing chamber, initially at a flow rate of 10 μl/min for 15 min and was later reduced to 1 μl/min. Intensity in counts per second was collected for 20 min or till the signals stabilized. Raw data was analyzed using BioAnalyst^TM^ software (AB Sciex Pte. Ltd., USA). Charge series (m/Z) peaks for monomer and dimer were converted into mass peaks using Bayesian peptide reconstruct tool, a built-in module of BioAnalyst^TM^ software.

### Arabidopsis protoplast preparation and transient transfections assay

Protoplast isolation and transfection were performed as described earlier. Details are given in supplementary data.

### RNA extraction and qRT- PCR analysis of transformed *Arabidopsis* protoplast

RNA was extracted from transformed protoplast by Trizol method. 2–5 × 10^5^ protoplasts were suspended in 0.5 ml TRIzol and β mercaptoethanol (0.01 part of TRIzol) and incubated at 65 °C for 10 min^42^. Protoplasts were centrifuged and supernatant was mixed with equal volume of chloroform:isoamyl alchohal (24:1), spun vigorously, and upper aqueous layer was removed and mixed with absolute ethanol and 1 M lithium chloride (100:1). Mixture was inverted and kept at −80 °C for 1 hour, thawed and washed with 70% ethanol and air dried at room temperature. Pellet was dissolved in TE buffer and quantified using Nanodrop spectrophotometer (Thermo Fischer Scientific, USA). cDNA was prepared as per manufacturer instructions (Invitrogen Superscript–III cDNA synthesis kit, USA). C_t_ values were normalized against ubiquitin^18^. Results were analyzed using comparative threshold cycle.

## Electronic supplementary material

Supplementary information
