# Supplementary material for: A-ZIP53, a dominant negative reveals the molecular mechanism of heterodimerization between bZIP53, bZIP10 and bZIP25 involved in Arabidopsis seed maturation
Source: Sci Rep. 2017 Oct 30;7:14343. doi: 10.1038/s41598-017-14167-5 (PMC5662769; doi:10.1038/s41598-017-14167-5)

Supplementary information

A-ZIP53, a dominant negative reveals the molecular mechanism of heterodimerization between bZIP53, bZIP10 and bZIP25 involved in *Arabidopsis* seed maturation.

Prateek Jain[1], Koushik Shah[1], Nishtha Sharma[1], Raminder Kaur[1], Jagdeep Singh[1], Charles Vinson[2] and Vikas Rishi[1*]

[1]National Agri-Food Biotechnology Institute, Knowledge City, Sector 81, Mohali, Punjab 140306, India

[2]Laboratory of Metabolism, National Cancer Institute, National Institutes of Health, MD 20892, USA

[*] To whom correspondence should be addressed.

Vikas Rishi, PhD
Scientist E
National Agri-Food Biotechnology Institute
(Department of Biotechnology, Government of India)
Room No 028, Ground Floor, NABI Research Building,
Knowledge City, Sector 81, Mohali
Punjab, India-140306
Tel: +91 172 5221123
Email: vikasrishi@nabi.res.in and vrishi09@gmail.com
www.nabi.res.in

Supplementary information-Protein sequences

Amino acids sequences of the full length proteins. Underlined sequences were cloned and expressed.

B-ZIP53
MGSLQMQTSPESDNDPRYATVTDERKRKRMISNRESARRSRMRKQKQLGDLINEVTLLKNDNAKITEQV
DEASKKYIEMESKNNVLRAQASELTDRLRSLNSVLEMVEEISGQALDIPEIPESMQNPWQMPCPMQPIRA
SADMFDC(Y_{18}–D_{116})-146

B-ZIP10
MNSIFSIDDFSDPFWETPPIPLNPDSSKPVTADEVSQSQPEWTFEMFLEEISSSAVSSEPLGNNNNAIVGV
SSAQSLPSVSGQNDFEDDSRFRDRDSGNLDCAAPMTTKTVIVDSDDYRRVLKNKLETECATVVSLRVGS
VKPEDSTSSPETQLQPVQSSPLTQGELGVTSSLPAEVKKTGVSMKQVTSGSSREYSDDEDLDEENETTG
SLKPEDVKKSRRMLSNRESARRSRRRKQEQTSDLETQVNDLKGEHSSLLKQLSNMNHKYDEAAVGNRIL
KADIETLRAKVKMAEETVKRVTGMNPMLLGRSSGHNNNNRMPITGNNRMDSSSIIPAYQPHSNLNHMSN
QNIGIPTILPPRLGNNFAAPPSQTSSPLQRIRNGQNHHVTPSANPYGWNTEPQNDSAWPKKCVD (V_{216}–
G_{301})-411

BZIP25
MHIVFSVDDLTESFWPVPAPAPSPGSSSTPSPTQNVADGMTRSQSEWAFHRLINELSGSDSSPTTNTIER
SPPPVQSLSRLEETVDETEDVVEIQKPQNHRRLPVDDQGKNRNRAPSSDPVDSSAPVVVDPNQYHAILK
SKLELACAAVARRVGTVKPEDSSASASNQKQAQGSIVAQTSPGASSVRFSPTTSTQKKPDVPARQTSISS
RDDSDDDDLDGDADNGDPTDVKRARRMLSNRESARRSRRRKQEQMNEFDTQVGQLRAEHSTLINRLS
DMNHKYDAAAVDNRILRADIETLRTKVKMAEETVKRVTGVNPLHWSRPNMGIPFSNTPSASSSIPPNSNHI
LKPANSSTNTSAGLAQNQRVETANFLPEQVNREGMQNPFAPDSNLYETLPHWNHKH (V_{230}-S_{322})-403

B-ZIP39
MVTRETKLTSEREVESSMAQARHNGGGGGENHPFTSLGRQSSIYSLTLDEFQHALCENGKNFGSMNMD
EFLVSIWNAEENNNNQQQAAAAAGSHSVPANHNGFNNNNNNGGEGGVGVFSGGSRGNEDANNKRGIA
NESSLPRQGSLTLPAPLCRKTVDEVWSEIHRGGGSGNGGDSNGRSSSSNGQNNAQNGGETAARQPTF
GEMTLEDFLVKAGVVREHPTNPKPNPNPNQNQNPSSVIPAAAQQQLYGVFQGTGDPSFPGQAMGVGD
PSGYAKRTGGGGYQQAPPVQAGVCYGGGVGFGAGGQQMGMVGPLSPVSSDGLGHGQVDNIGGQYG
VDMGGLRGRKRVVDGPVEKVVERRQRRMIKNRESAARSRARKQAYTVELEAELNQLKEENAQLKHALA
ELERKRKQQYFESLKSRAQPKLPKSNGRLRTLMRNPSCPL(V_{351}–L442)-442

B-ZIP62
MELEPISSSCCSSSSSSSGEENTAAANMTEMEAAEALADLAQLAIMREQVFESAASWGSKGKRVRKRVK
TESPPSDSLLKPPDSDTLPTPDLAEERLVKEEEEEEEVEPITKELTKAPVKSEINGETPKPILASTLIRCSRS
NGCGRSRQNLSEAEREERRIRRILANRESARQTIRRRQAMCEELSKKAADLTYENENLRREKDWALKEF
QSLETINKHLKEQVLKSVKPDTKEPEESPKPSQVEMSTSSTPFYFYNQNPYQLFCWPHVTQSSNPMISPL
EFPTSGGASAKTITTQEHENAADDNGQKTHFYVVPCPWFLPPPDHSNGVPFGLQDTQRGTFSNGHHIDD
SSARPMDVTETPRSHLPTRIKEEDSGSPETRPLYDLNESATEVLSEGGDGFPVTQQAYSLKHEDVSETTN
GVTLMPPGHHVLISLPEKKHGSLAAAEARKRRKELTRLKNLHGRQCRMQVG (E_{156}-S_{251})-471

B-ZIP72
MSFPVVATSFGVSQSGSQAGKKKGGYVNYEVEPGFTIRMRQNIDPTTDPKILKRIISNRVAAQKSRWKKV
QYLDALVKRSMELQREVSELRSQLAITSEQKRYLENEQRQLKECISARVQHCINSDGVIEEYKTEIERLKT
NLAPLSNLT (D_{48}-T_{150})-150

A-ZIP39
LEQRAEELARENEELEKEAEELEQELAELENELNQLKEENAQLKHALAELERKRKQQYF
ESLKSRAQPKLPKSNGRLRTLMRNPSCPL

A-ZIP62
LEQRAEELARENEELEKEAEELEQELAELENKAADLTYENENLRREKDWALKEFQSLETINKHLKEQVLK SVK

Supplementary Table S1:

Primers for cloning *Arabidopsis thaliana* B-ZIP transcription factors as BamHI-HindIII insert

SNo.	Gene	primer	Sequence (**C** is added in the forward primer after the BamHI site to keep protein in Frame)
1	B-ZIP10 (At4g02640)	Forward	GACGG**GGATCC**CGTTAAAAAATCTAGAAGGATG
		Reverse	GACGGC**AAGCTT**TTATCCGAGAAGCATCGGATTC
2	B-ZIP25 (At3g54620)	Forward	GACGG**GGATCC**CGTGAAGCGTGCTAGGAGGATG
		Reverse	GACGGC**AAGCTT**TTATGACCAATGCAAAGGGTTC
3	B-ZIP39 (At2g36270)	Forward	GACGG**GGATCC**CGTGGAGAAAGTAGTGGAGAG
		Reverse	GACGGC**AAGCTT**TTAGAGTGGACAACTCGGG
4	B-ZIP53 (At3g62420)	Forward	GACGG**GGATCC**CAGGTACGCCACGGTGACGG
		Reverse	GACGGC**AAGCTT**TTAATCCAAAGCCTGACCACTAA
5.	B-ZIP62 (At1g19490)	Forward	GACGG**GGATCC**CGAAAGAGAAGAACGTAGAATC
		Reverse	GACGGC**AAGCTT**TTAAGATGATGTAGACATCTCA
6	Removing internal	Forward	GTATTTCTTTGACGCTTCATCAAC
	HindIII site in B-ZIP53	Reverse	GTTGATG**AAGCGT**CAAAGAAATAC

Primers for synthesizing B–ZIP72

1	Primer 1	GACGGGGATCCCCGTATGCGTCAGAATATTGATCCCACCACGGACC CTAAGATACTCAAACGGATAATTTCGAACCGTG
2	Primer 2	GATCTCTTCACTAGAGCATCAAGATATTGAACCTTCTTCCACCTAGAC TTTTGAGCCGCAACACGGTTCGAAATTATCCG
3	Primer 3	GATGCTCTAGTGAAGAGATCCATGGAGCTCCAAAGAGAGGTGAGTG AGTTGCGTTCCCAACTAGCGATTACCAGTGAAC
4	Primer 4	GAACCCGAGCAGAGATACACTCCTTTAGCTGCCTTTGCTCATTTTCT AGATATCGTTTTTGTTCACTGGTAATCGCTAG
5	Primer 5	GTGTATCTCTGCTCGGGTTCAACATTGTATCAACAGCGATGGTGTCA TTGAGGAGTATAAAACGGAGATAGAG

6	Primer 6	GACGGCAAGCTTCTAAGTAAGATTGGAGAGTGGAGCCAGATTCGTC TTCAGCCTCTCTATCTCCGTTTTATACTCC	
Primers for cloning A-ZIP53 and its derivatives.			
1	A-ZIP53	Forward	GACGGC**TCGAG**AACGAAGTCACTCTTCTC
2		Reverse	GACGGC**AAGCTT**TTAATCCAAAGCCTGACCACTA A
3	Common primers for cloning derivatives of A-ZIP53	Forward	GACGG**GGATCC**TGACCTGGAACAA
		Reverse	GACGG**CTCGAG**TTCTGCCAGTTCCTG
4	A→E	Forward	GAGGAACTGGAGCGTGAAAAC
		Reverse	GTTTTCACGCTCCAGTTCCTC
5	N→A	Forward	GCCCGTGAAGCCGAAGAGCTG
		Reverse	CAGCTCTTCGGCTTCACGGGC
6	R→E	Forward	GGTCG**GGATCC**TGACCTGGAACAAGAGGCTGAG GAACTGGCC
		Reverse	GACGGCTCGAGTTCTGCCAGTTCCTG
6	A→E, N→A	Forward	GAACTGGAGCGTGAAGCCGAAGAG
		Reverse	CTCTTCGGCTTCACGCTCCAGTTC
Sequencing primers for pT5 plasmid			
1		Upstream	GGGAGACCACAACGGTTTCCCTC
2		Downstream	ATGCTAGTTATTGCTCAGC

Primers for the A-ZIP39 and A-ZIP62

1	A-ZIP39	Forward	GACGG**CTCGAG**GCTGAACTTAACCAGTTG
		Reverse	GACGGC**AAGCTT**TTAGAGTGGACAACTCGGG
2	A-ZIP62	Forward	GACGG**CTCGAG**AAAAAAGCAGCTGATCTG
		Reverse	GACGGC**AAGCTT**TTAAGATGATGTAGACATCTCA

Methods

Protein expression and purification

All proteins used here contain N-terminus 13 amino acids T7 tag (ASMTGGQQMGRDP). B-ZIPs and A-ZIPs expressing plasmids were transformed into *E. coli* BL21 (DE-3) strain following standard protocols (1). 20 ml of *E. coli* cultures containing ampicillin (100 µg/ml) and chloramphenicol (25 µg/ml) were grown overnight and transferred to 500 ml of pre-warmed Superbroth medium containing 100 µg/ml of ampicillin at 37° C and shaken continuously at 300 rpm till O.D. reached 0.6 at 600 nm. *E. coli* were induced with 1 mM Isopropyl-D-thiogalactopyranoside (IPTG, Sigma, USA) and further grown for 3-5 hours (1). Bacteria were pelleted down by centrifugation at 4000 rpm and lysed by low salt lysis buffer and immediately frozen at -20° C. Bacteria were thawed at room temperature in the presence of 1 M KCl. This step releases DNA-bound B-ZIP protein in the solution and also precipitates bacterial DNA. High-salt protein suspension was heated to 65° C for 15 minutes cooled at room temperature and debris was centrifuged at 20,000 rpm. At this stage B-ZIPs and A-ZIPs are close to 70% pure. Samples were dialyzed overnight against dialysis buffer (20 mM Tris-HCl (pH 8.0), 50 mM KCl, 1 mM EDTA, 0.2 mM phenylmethylsulfonyl fluoride (PMSF), and 1 mM DTT). Dialyzed B-ZIP samples were loaded on to hepharin-sepharose column (GE Healthcare, USA) and bound proteins were eluted using potassium phosphate buffer (pH 7.4) with increasing salt concentrations (50 mM, 150 mM, 300 mM, and 1M KCl). 300 mM and 1 M fractions were pooled and were subjected to Reverse Phase-High Pressure Liquid Chromatography (RP-HPLC) purification. For A-ZIP proteins hydroxyapatite (Bio-Rad., USA) columns were used. Dialyzed samples of A-ZIP proteins were loaded on to Hydroxyapatite columns and resin-bound proteins were eluted with 250 mM sodium phosphate buffer (pH 7.4) and were finally purified by RP-HPLC. For RP-HPLC, C_{18} hydrophobic column (Agilent, USA) was used. All solution contained 0.1% TFA as an ion-pair agent. Gradient of degassed solvent A (distil water with 0.1% TFA) and solvent B (100% acetonitrile with 0.1% TFA) was used for final purification of B-ZIP and A-ZIP proteins. Gradient of 0-100% of solvent B (achieved in 45 minutes with flow rate of 2 ml/min) was used to elute proteins from the column. Peak absorbance was monitored at 220 nm. HPLC purified protein samples were lyophilized and dissolved in the CD buffer (12.5 mM phosphate buffer (pH 7.4), 150 mM KCl, and 0.25 mM EDTA) and concentration was measured as described earlier.

Circular dichroism (CD) spectroscopy

All circular dichroic (CD) studies were carried out using 815 spectropolarimeter (Jasco, Japan). Samples were prepared in the standard CD buffer. Before CD studies, samples were heated to 65° C for 15 min and allowed to cool at room temperature. For structural studies, 2 µM homodimer and 4 µM heterodimer proteins were used. Wavelength scans of proteins and DNA were carried at 6° C and samples were scanned from 200-260 nm. Average of 5 scans for each protein sample was taken. For thermal denaturation studies protein samples were heated from $6\text{-}75/85^o$ C at the rate of 1° C/min while change in

ellipticity was measured at 222 nm. Leucine zipper of all B-ZIP proteins alone or as heterodimers with A-ZIPs and A-ZIP53 (A→E) derivative melted with well-defined transition curve. All such curves were fitted according to following equation assuming denaturation to be a two-state type (2)

$$-\theta_{222}(T) = (N - D)(1 - T/T_{inter})\,[1 + 1 - (8\exp(\Delta H_m(1/T_m - 1/T)/\mathbf{R} + 1))^{1/2} / 4\exp \Delta H_m(1/T_m - 1/T)/\mathbf{R}] + D \qquad (1)$$

where $-\theta_{222}(T)$ were CD signals in mdeg at 222 nm at any temperature T in ^{o} C, N is the ellipticity of the α-helical coiled coil dimer at 0° C extrapolated from the linear-dependencies of CD signals at low temperature range defined as pre-transition region, D is the ellipticity at high temperature extrapolated to 0° C and referred to as post-transition baseline where dimeric protein molecules melt to unhelical monomers. Transition region between pre-and post-transition region has monomer and dimer B-ZIPs in equilibrium. T_{inter} is the temperature at which the linear temperature-dependencies of dimer and monomer molecules intersect, and **R** is the gas constant. Fitting of thermal denaturation curves gave values of midpoint of thermal denaturation (T_m), and enthalpy change at T_m (ΔH_m). Constant-pressure heat capacity change (ΔC_p) was calculated using T_m versus ΔH_m plot for all the homo- and heterodimer B-ZIPs and A-ZIP53 (A→E) protein. A linear fitting of these data points with negative slope gave the value of ΔC_p = -1.73 ± 0.18 kcal mol^{-1} dimer K^{-1}(supplementary Figure S1). This along with T_m, and ΔH_m values were used to calculate ΔG_{Di}, Gibbs free energy of dimerization at 25° C for each dimer by using the following form of Gibbs-Helmholtz equation (2).

$$\Delta G_{Di} = \mathbf{R}T_o \ln C + \Delta H_m(1-T_o/T_m) - \Delta C_p[(T_m - T_o) + T_o \ln(T_o/T_m) \qquad (2)$$

where T_o is any temperature in ^{o} C and C is total protein concentration. ΔG_{Di} values are included in Table 2.

Arabidopsis protoplast preparation and transient transfections assay

Healthy leaves from the four weeks old *Arabidopsis* were taken for the protoplast isolation. 0.5 mm-1 mm leaf sections were cut from the middle with sharp razor blade. For 10^{6}-10^{7} protoplasts approximately 40-50 leaves were used. Cut sections were digested in 10 ml of enzyme solution (20 mM MES (pH 5.7) containing 1.5% (wt/vol) cellulase (Sigma, USA), 0.4 % (wt/vol) macerozyme (Sigma, USA), 0.4 M mannitol, and 20 mM KCl). Inactivation of endogenous leaf DNAse and protease was achieved by heating the solution at 55° C for 10 min. Solution was cooled at room temperature and 10 mM $CaCl_2$, 3 mM β-mercaptoethanol, and 0.1 % BSA were added. Leaves were vacuum infiltrated for 30 min in dark using desiccator. Digestion was continued by putting the leaves in dark for 3-4 hours at room temperature. Release of protoplasts was marked by solution turning green. Intact protoplasts were checked under the microscope. Enzyme solution was diluted with equal volume of W5 solution (2 mM MES (pH 5.7)

containing 154 mM NaCl, 125 mM $CaCl_2$, and 5 mM KCl). Debris was removed by filtration using muslin cloth. Solution was centrifuged at 100 g in a 30 ml round bottomed tube for 1 min, supernatant removed and pellet was re-suspended at 2 X 10^5 cells density in W5 solution. Protoplast were kept in ice for 30 min and allowed to settle. W5 solution was removed without disturbing protoplast pellet. Protoplasts were resuspended at 2 X 10^5 /ml density in MMG solution (4 mM MES (pH 5.7) containing 0.4 M mannitol and 15 mM $CaCl_2$) (3).

For transient transfection studies, 9 µg plasmid overexpressing either B-ZIP53 or B-ZIP10 or B-ZIP25 and their equimolar mix in three combinations (B-ZIP53+B-ZIP10, B-ZIP53+B-ZIP25, B-ZIP25+B-ZIP10) and 14 µg Camv35S:A-ZIP53 plasmid, 9 µg of GUS reporter plasmid, and 1 µg NAN coding plasmid (an internal control for transfection efficiencies) were mixed with 100 µl of protoplast solution containing ~2 x10^4 protoplasts and mixed gently. 110 µl of PEG solution (20-40% (wt/vol, PEG3500 in double distilled water containing 200 mM mannitol, and 100 mM $CaCl_2$) was added (4,5). Transfection mixture was incubated at room temperature for 15 min and subsequently diluted with 400 µl of W5 solution and gently inverted. Protoplasts were re-suspended in 1 ml W1 solution (4 mM MES (pH 5.7), 0.5 M mannitol, and 20 mM NaCl) divided into six tubes. Protoplasts were incubated in dark at room temperature (20 - 25° C) for 16-18 hours. Protoplast suspensions were centrifuged at 100 g for 2 min at room temperature. Supernatant was removed and analyzed for GUS/NAN activities (6,7).

References

1. Rishi, V., Gal, J., Krylov, D., Fridriksson, J., Boysen, M.S., Mandrup, S. and Vinson, C. (2004) SREBP-1 dimerization specificity maps to both the helix-loop-helix and leucine zipper domains: use of a dominant negative. *The Journal of biological chemistry*, **279**, 11863-11874.
2. Krylov, D., Mikhailenko, I. and Vinson, C. (1994) A thermodynamic scale for leucine zipper stability and dimerization specificity: e and g interhelical interactions. *EMBO J.*, **13**, 2849-2861.
3. Yoo, S.D., Cho, Y.H. and Sheen, J. (2007) Arabidopsis mesophyll protoplasts: a versatile cell system for transient gene expression analysis. *Nat Protoc*, **2**, 1565-1572.
4. Ehlert, A., Weltmeier, F., Wang, X., Mayer, C.S., Smeekens, S., Vicente-Carbajosa, J. and Droge-Laser, W. (2006) Two-hybrid protein-protein interaction analysis in Arabidopsis protoplasts: establishment of a heterodimerization map of group C and group S bZIP transcription factors. *Plant J.*, **46**, 890-900.
5. Weltmeier, F., Ehlert, A., Mayer, C.S., Dietrich, K., Wang, X., Schutze, K., Alonso, R., Harter, K., Vicente-Carbajosa, J. and Droge-Laser, W. (2006) Combinatorial control of Arabidopsis proline dehydrogenase transcription by specific heterodimerisation of bZIP transcription factors. *EMBO J.*, **25**, 3133-3143.
6. Alonso, R., Onate-Sanchez, L., Weltmeier, F., Ehlert, A., Diaz, I., Dietrich, K., Vicente-Carbajosa, J. and Droge-Laser, W. (2009) A pivotal role of the basic leucine zipper transcription factor

bZIP53 in the regulation of Arabidopsis seed maturation gene expression based on heterodimerization and protein complex formation. *Plant Cell*, **21**, 1747-1761.

7. Kirby, J. and Kavanagh, T.A. (2002) NAN fusions: a synthetic sialidase reporter gene as a sensitive and versatile partner for GUS. *Plant J.*, **32**, 391-400.

LEGENDS TO SUPPLEMENTARY FIGURES:

Supplementary Figure S1: Temperature-dependence of enthalpy change. Slope of the linear fit of the plot of ΔH_m Vs T_m obtained from the analysis of CD thermal transitions curves for all the proteins used in this study gave the value of ΔC_p. The value ΔC_p was used to obtain ΔG_{Di} for all the proteins used here. The assumption is that the major contribution to temperature-dependence of ΔH_m is ΔC_p.

Supplementary Figure S2: ESI-MS spectrum showed that in the aqueous solution, B-ZIP53 and A-ZIP53 exist as dimer and monomer. Experiments were performed at 22 ± 2° C. **A**) ESI-mass spectra of 2 µM of B-ZIP53 in MS grade water. Charge series +8 - +13, and +22, +23, +25, correspond to the monomer and dimer B-ZIP53 respectively. **B**) Mass spectra of B-ZIP53 obtained from **A**. B-ZIP53 predominately exist as monomer (MW 12957) and dimer (MW 25914) with minor peaks that corresponds to truncated dimers. **C**) ESI mass spectra of 2 µM of A-ZIP53 in MS grade water. Charge series +8 - +10, and +14 - +16 indicates monomer conformation whereas +22 - +26 charge series corresponds to dimer conformation. **D**) Molecular mass spectra obtained from **C** showed A-ZIP53 as monomer (=12221 Da) and dimer (=24442 Da).

Supplementary Figure S3: **A)** Alignment of acidic helical extensions used in this study with DNA-binding region of the wild-type B-ZIP10 and B-ZIP25, showing the possible interhelical interactions in homodimers and heterodimers. At the top is shown the coiled-coil heptad designations (*a,b,c,d,e,f*, and *g*) is shown. **B)** Coiled coil helical wheel diagram of the interactions between B-ZIP10 and B-ZIP25 with the A-ZIP53 looking from the N-terminus. The coiled coil sequence reads outward from N- to C-termini around the wheel, starting at the g position of L_{-3}. Possible electrostatic interactions between *g*↔*e*' and *g*↔*a*' are shown. In the box, the changed amino acids in derivatives of A-ZIP53 DN are shown.

Supplementary Figure S4: Transcript levels of all B-ZIPs used here at different development stages of seed development and maturation. Gene investigator data revealed the expression of B-ZIP53, B-ZIP10, B-ZIP25, B-ZIP39, B-ZIP62, and B-ZIP72 in the different developmental stages of *Arabidopsis* including during seed development.

Supplementary Figure S5: CD thermal denaturation profiles of A-ZIP53 (A→E), A-ZIP53 (R→E), and their heterodimer with three B-ZIPs used here. **A**) Thermal denaturations of 2 µM each of A-ZIP53

(A→E), B-ZIP53 and their equimolar mixture (4 µM). An increase in the thermal stability of A-ZIP53 (A→E)|B-ZIP53 protein sample suggests that a stable heterodimer was formed. **B**) Same as in **A** except that B-ZIP10 was melted with A-ZIP53 (A→E). **C**) Same as **A** except that B-ZIP25 was melted alone and as heterodimer with A-ZIP53 (A→E). **D-F**) Same as **A**. As shown, A-ZIP (R→E), a R→E version of A-ZIP53 was melted alone and as heterodimer with B-ZIP53 **D**), B-ZIP10 **E**), and B-ZIP25 **F**). In all the cases stable heterodimers were formed as indicated by increase in T_m values of the heterodimers.

Supplementary Figure S6: CD thermal denaturation profiles of A-ZIP53 (N→A), A-ZIP53 (A→E, N→A), and their heterodimer with three B-ZIPs used here. **A**) Thermal denaturation of 2 µM dimer each of A-ZIP53 (N→A), B-ZIP53, and their equimolar mix. An increase in the thermal stability of A-ZIP53 (N→A)|B-ZIP53 protein sample suggests that a stable heterodimer was formed. **B**) Same as **A** except that B-ZIP10 was melted with A-ZIP53 (N→A). **C**) Same as **A** except that B-ZIP25 was melted alone and as heterodimer with A-ZIP53 (N→A). **D-F**) Similar to **A**. As shown, double mutant A-ZIP53 (N→A, A→E) was melted alone and as heterodimer with B-ZIP53 (**D**), B-ZIP10 (**E**), and B-ZIP25 (**F**). In all cases stable heterodimers were formed indicated by increased T_m.

Supplementary Figure S1

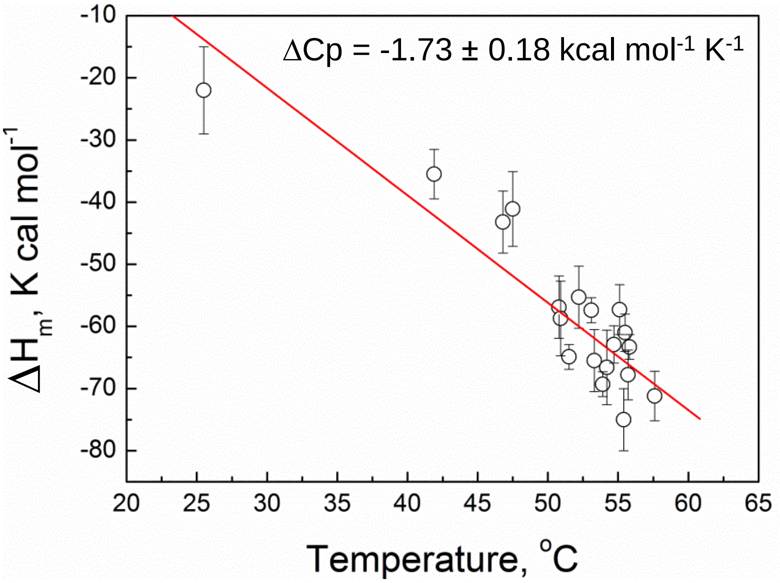

Supplementary figure S2

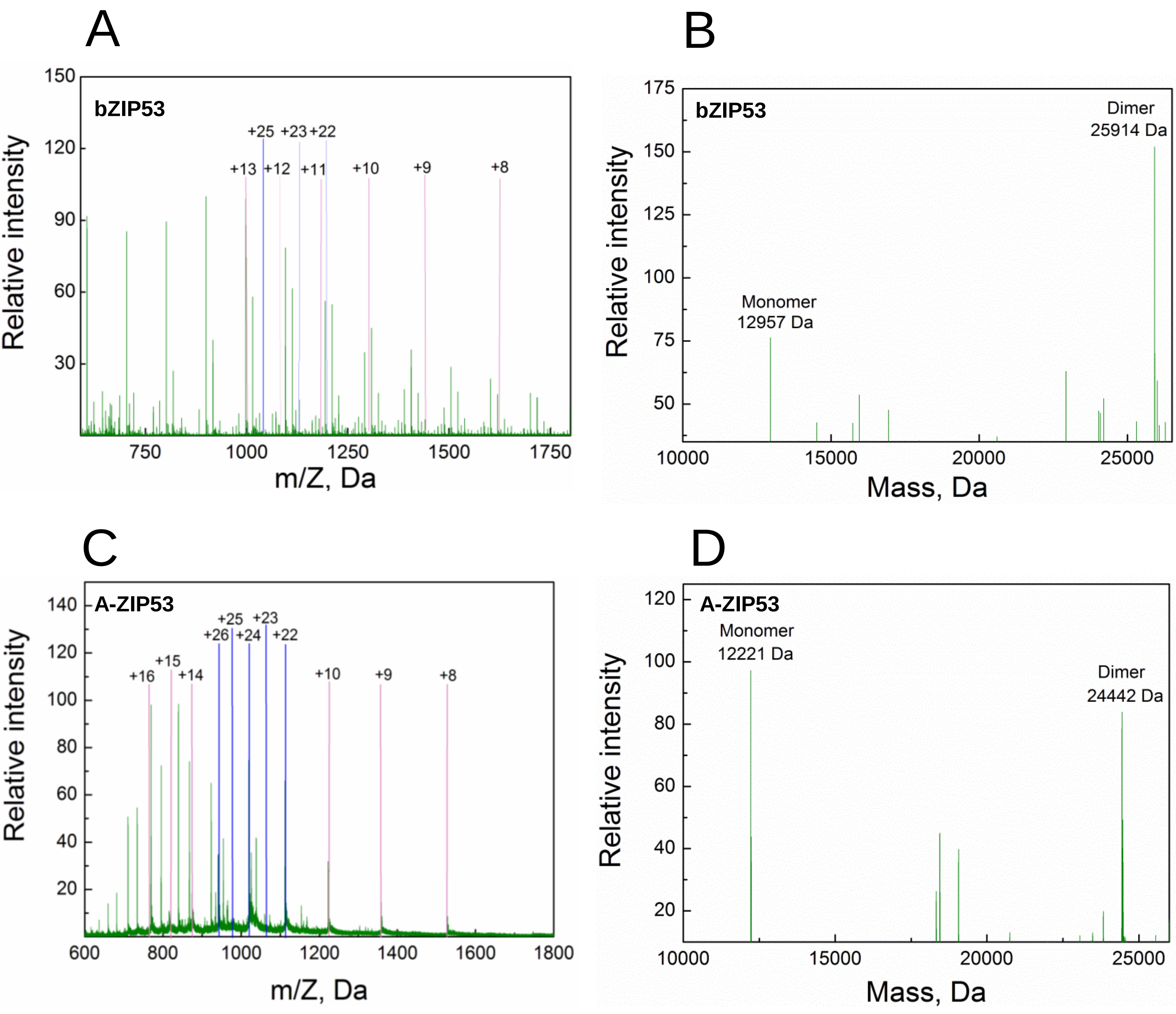

Supplementary Figure S3

A

```
                 L-3      L-2      L-1      Lo
          def gabcdef gabcdef gabcdef gabcdef
bZIP10    PVK KSRRMLS NRESARR SRRRKQE QTSDLET
A-ZIP53   LEQ RAEELAR ENEELEK EAEELEQ ELAELEN

bZIP25    PVK RARRMLS NRESARR SRRRKQE QMNEFDT
A-ZIP53   LEQ RAEELAR ENEELEK EAEELEQ ELAELEN
```

B

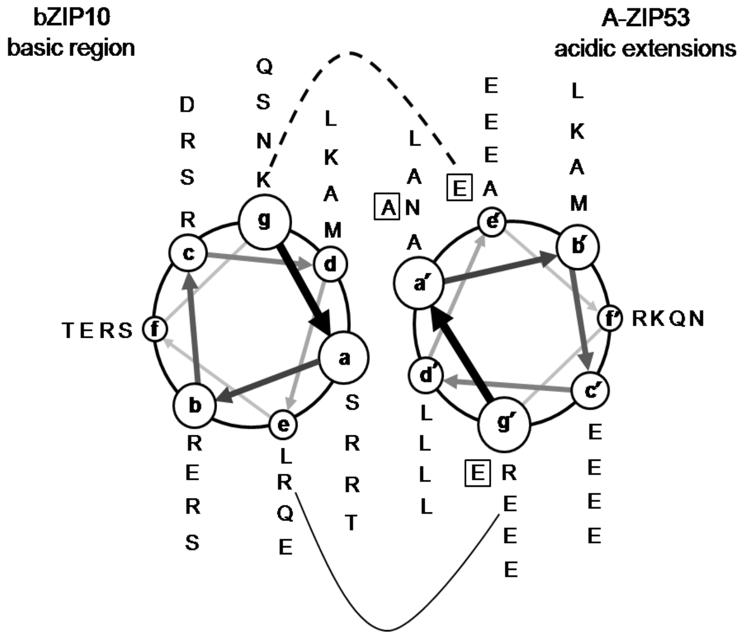

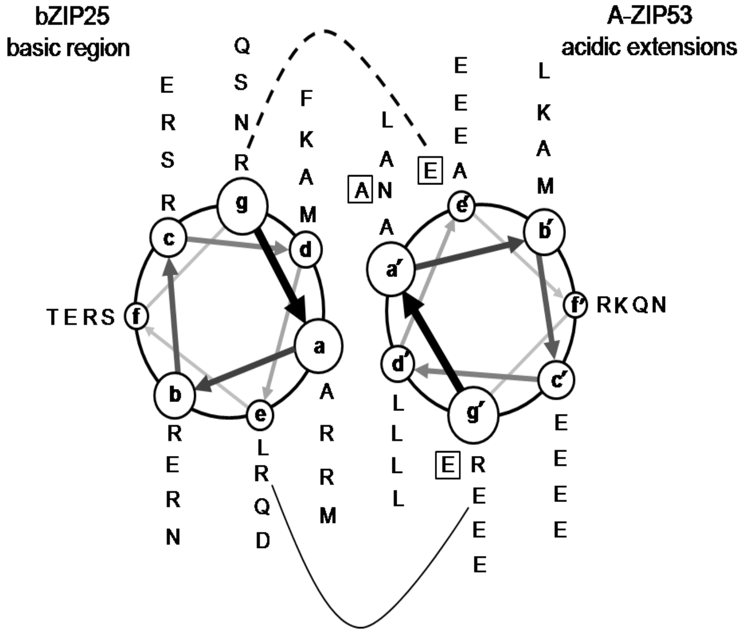

Supplementary Figure S4

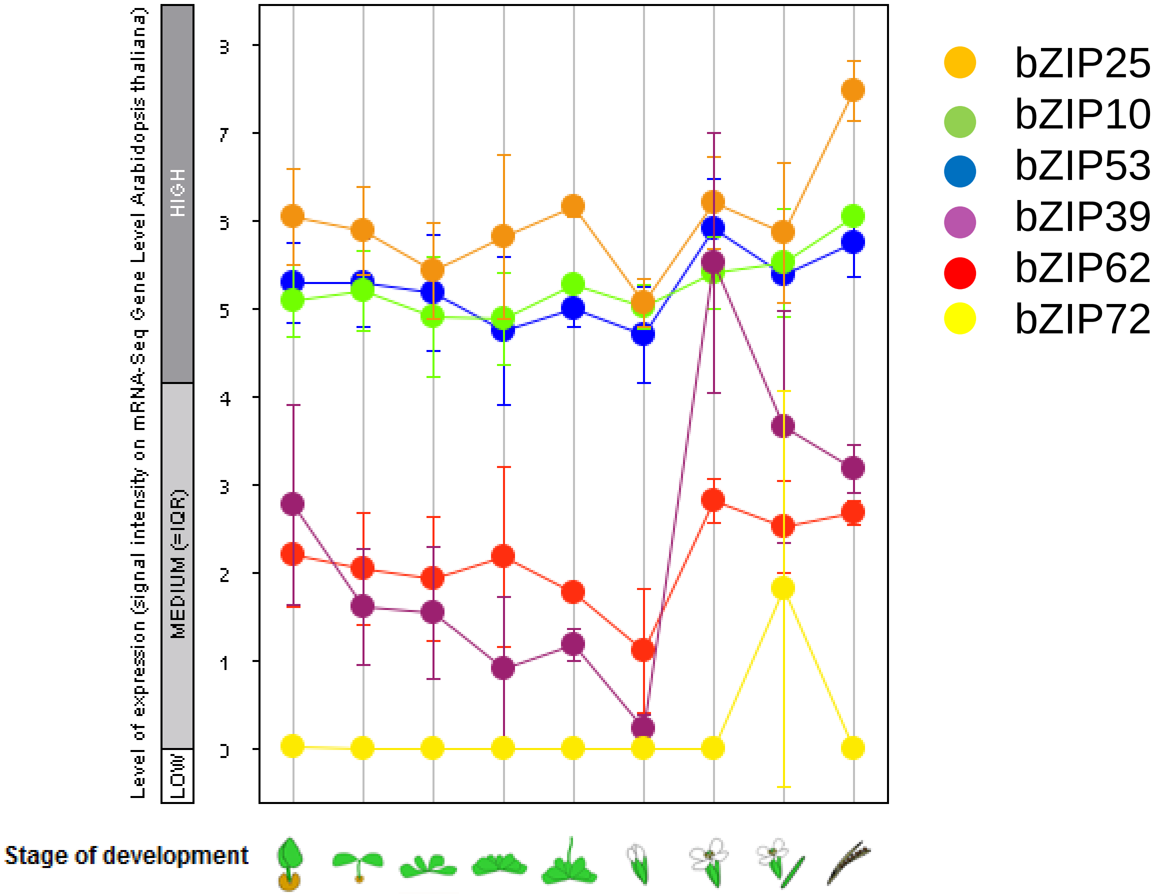

Supplementary Figure S5

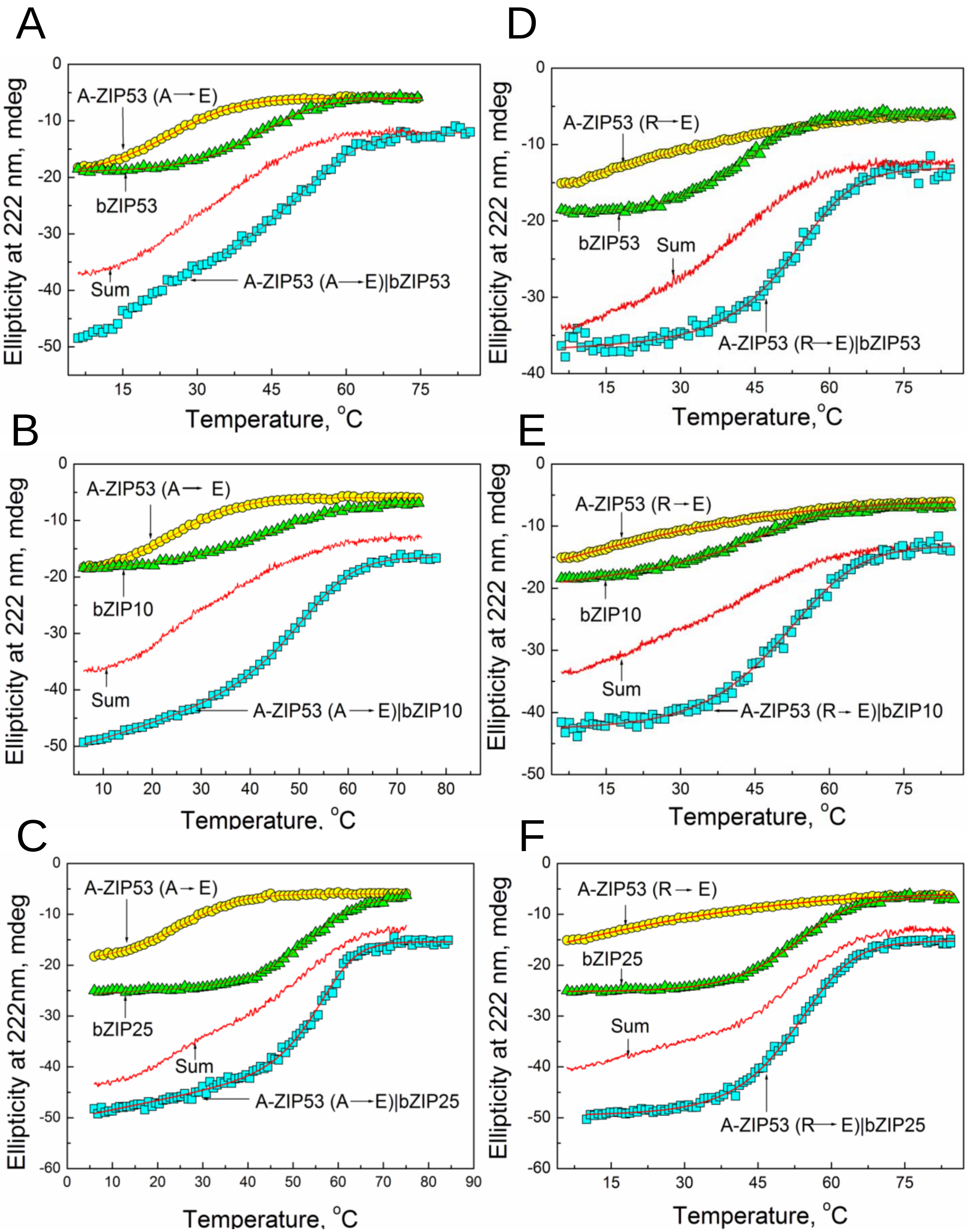

Supplementary Figure S6

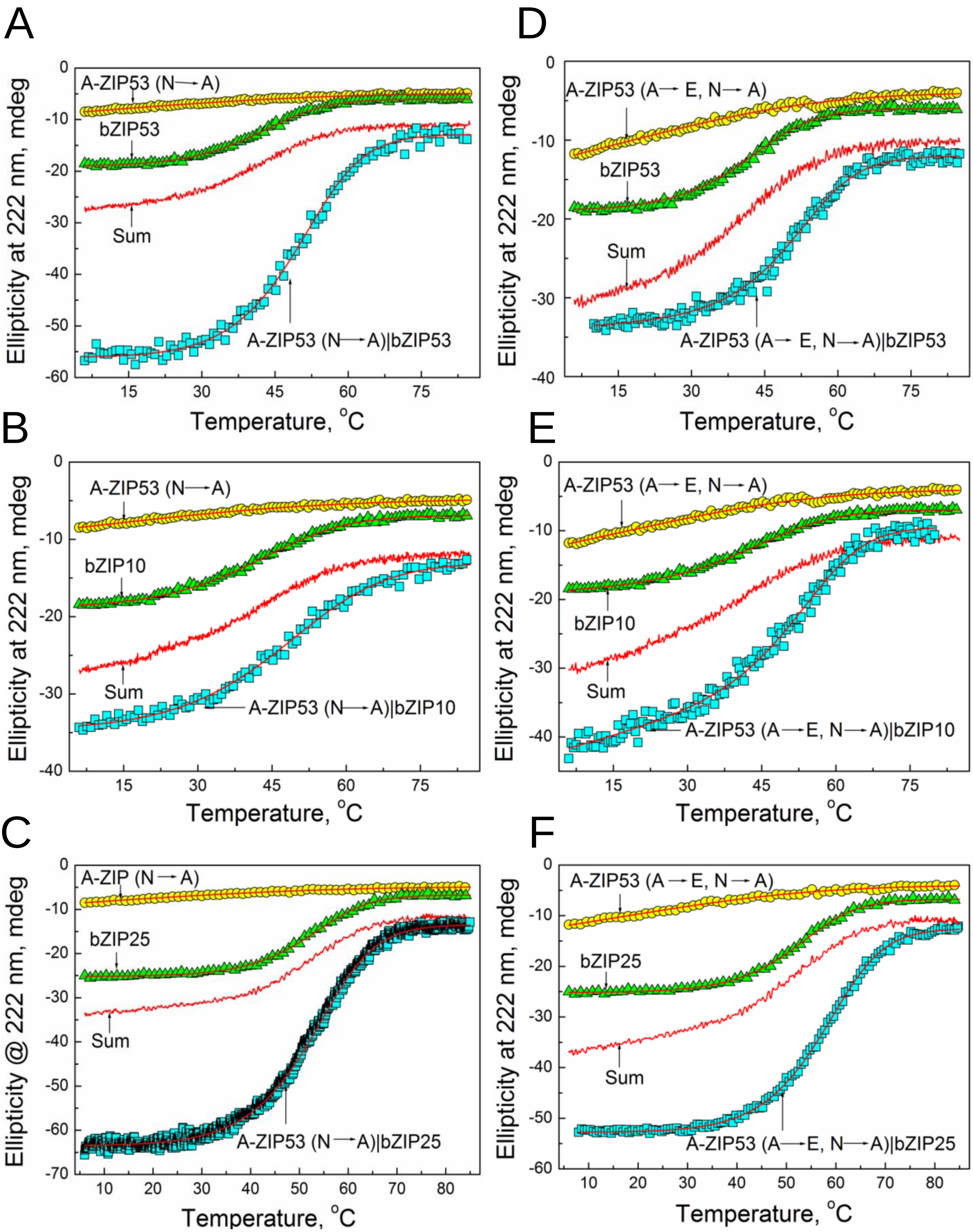

Supplement: Supplementary file 1 — Supplementary information [file 41598_2017_14167_MOESM1_ESM.pdf]
